# A new chemical inhibitor of angiogenesis and tumorigenesis that targets the VEGF signaling pathway upstream of Ras

**DOI:** 10.18632/oncotarget.2979

**Published:** 2015-01-23

**Authors:** Agnès Desroches-Castan, Delphine Quélard, Martine Demeunynck, Jean-François Constant, Chongling Dong, Michelle Keramidas, Jean-Luc Coll, Caroline Barette, Laurence Lafanechère, Jean-Jacques Feige

**Affiliations:** ^1^ Institut National de la Santé et de la Recherche Médicale (INSERM), Unité 1036, Biology of Cancer and Infection, Grenoble, F-38054, France; ^2^ Univ. Grenoble-Alpes, Department of Chemistry, Biology and Health Sciences, Grenoble, F-38000, France; ^3^ Commissariat à l'Energie Atomique (CEA), DSV/iRTSV, Grenoble, F-38054, France; ^4^ Janssen, Pharmaceutical Companies of Johnson and Johnson, Issy-les-Moulineaux, F-92130, France; ^5^ Centre National de la Recherche Scientifique (CNRS), UMR 5063, Department of Molecular Pharmacochemistry, Grenoble, F-38041, France; ^6^ Centre National de la Recherche Scientifique (CNRS), UMR 5250, Department of Molecular Chemistry, Grenoble, F-38041, France; ^7^ Institut National de la Santé et de la Recherche Médicale (INSERM), Unité 823, Albert Bonniot Research Center, La Tronche, F-38700, France; ^8^ Institut National de la Santé et de la Recherche Médicale (INSERM), Unité 1038, Large Scale Biology, Grenoble, F-38054, France

**Keywords:** angiogenesis, chemical library, VEGF signaling, drug development

## Abstract

The efficacy of anti-angiogenic therapies on cancer patients is limited by the emergence of drug resistance, urging the search for second-generation drugs. In this study, we screened an academic chemical library (DCM, University of Grenoble-Alpes) and identified a leader molecule, COB223, that inhibits endothelial cell migration and proliferation. It inhibits also Lewis lung carcinoma (LLC/2) cell proliferation whereas it does not affect fibroblast proliferation. The anti-angiogenic activity of COB223 was confirmed using several *in vitro* and *in vivo* assays. In a mouse LLC/2 tumor model, ip administration of doses as low as 4 mg/kg COB223 efficiently reduced the tumor growth rate. We observed that COB223 inhibits endothelial cell ERK1/2 phosphorylation induced by VEGF, FGF-2 or serum and that it acts downstream of PKC and upstream of Ras. This molecule represents a novel anti-angiogenic and anti-tumorigenic agent with an original mechanism of action that deserves further development as an anti-cancer drug.

## INTRODUCTION

It is now well accepted that angiogenesis is a rate-limiting step in tumor progression [1]. Deciphering the molecular mechanisms of tumor angiogenesis has recently allowed successful translation into clinical applications. Novel classes of anticancer drugs that specifically target tumor endothelial cells have been developed and several clinical trials have demonstrated their benefits in the treatment of distinct types of metastatic cancers [2, 3]. These include the neutralizing anti-VEGF antibody bevacizumab, the soluble binding domain of VEGF receptors aflibercerpt and several small tyrosine kinase inhibitors targeting VEGF receptor and other kinases, including sorafenib, sunitinib, pazopanib and regorafenib. However, up to now, none of the anti-angiogenic drugs that were approved on the pharmaceutical market can irreversibly stop tumor progression and turn back cancer tumors into their dormant state. The major problems encountered with anti-angiogenic drugs are that a hardly predictable but consistent percentage of patients are or become resistant to these therapies [2, 4]. There is thus a strong need to develop second-generation anti-angiogenic drugs that could be used in second line in resistant patients.

In this work, we screened the recently built-up library of the Department of Molecular Chemistry from the University of Grenoble (France) for compounds that can efficiently interfere with the angiogenic process. Such phenotypic screens - in which a compound collection is directly assayed on cells - are extremely effective to discover drugs with new mechanism of action without any *a priori* on their biological target. Indeed, a recent survey has established that the vast majority of first-in-class drugs were identified by phenotypic screening [5]. Although small (1360 molecules), this library presents the advantage of containing original non-commercial molecules. We adapted the endothelial cell scratch assay to the 96-well microplate format, since this assay correlates well with the *in vivo* angiogenic response [6]. Using this assay, we selected a family of polyamine derivatives that potently inhibit endothelial wound healing. We further assessed their anti-angiogenic and anti-tumorigenic potencies in several *in vitro* and *in vivo* assays. We also succeeded at identifying the mechanism of action of the leader molecule that appears to inhibit the Ras/Raf/ERK pathway upstream of Ras and downstream of the activated growth factor receptors.

## RESULTS

### Setting up the HTS screening assay

**Figure 1:**
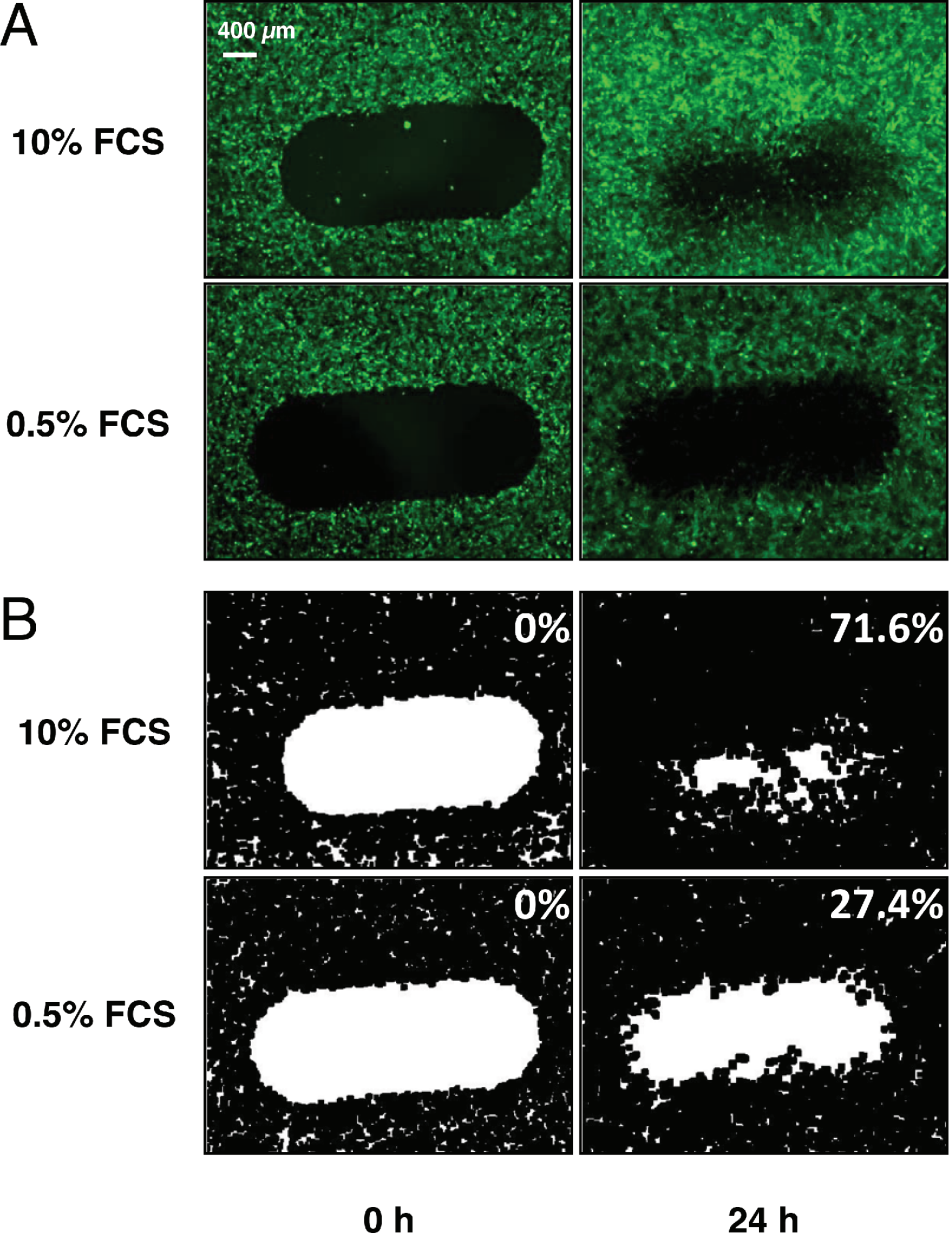
Automatization of the endothelial wound closure assay Monolayers of fluorescent HMEC-GFP cells were grown in 96 well-plates and automatically scratched by the HTS robot. Cells were then incubated for 24 h in the presence of 10% FCS and the various molecules to be tested. The negative (0.5% FCS alone) and positive (10% FCS alone) controls are also presented. **(A)** fluorescence microscopy imaging of wound closure at time 0 h (left column) and 24 h (right column). **(B)** Numerization of the images as indicated in Material and Methods allowing quantification of % of wound closure (indicated in the upper right corners of each panel).

In order to adapt the endothelial scratch wounding assay to the constraints of high throughput screening, we decided to use an endothelial cell line rather than primary cultures for the sake of reproducibility. Since angiogenesis is triggered by capillary endothelial cells, we selected the HMEC-1 cell line which consists of human dermal microvascular endothelial cells stably expressing SV40 middle T antigen [7]. We infected this cell line with GFP-encoding retroviral particles and selected one clone (thereafter named HMEC-GFP) that presented a high level of fluorescence and a strong proliferation rate (doubling time in the presence of serum: 22 h). We controlled that GFP expression was stable over at least 50 passages and that these cells still expressed endothelial markers such as VE-cadherin and CD31. In a standard assay, HMEC-GFP cells were grown until confluence in 96-well plates in the presence of serum, scratched with the multi-tip dispenser of the HTS automat, gently rinsed twice to eliminate detached cells and cell debris that could liberate inflammatory and potentially angiogenic cytokines, and incubated for 24 h in the presence of serum and the compounds to be tested. Each well was photographed at time 0 h (after wounding) and 24 h under a motorized epifluorescence microscope and the digitized images were analyzed using Image J to determine the percentage of wound closure (Figure 1A, 1B). Under our standard conditions, the closure rate was 72 ± 4% in the positive controls and 27 ± 4.% in the negative controls.

### Identification of polyamine derivatives as inhibitors of endothelial cell migration

**Figure 2:**
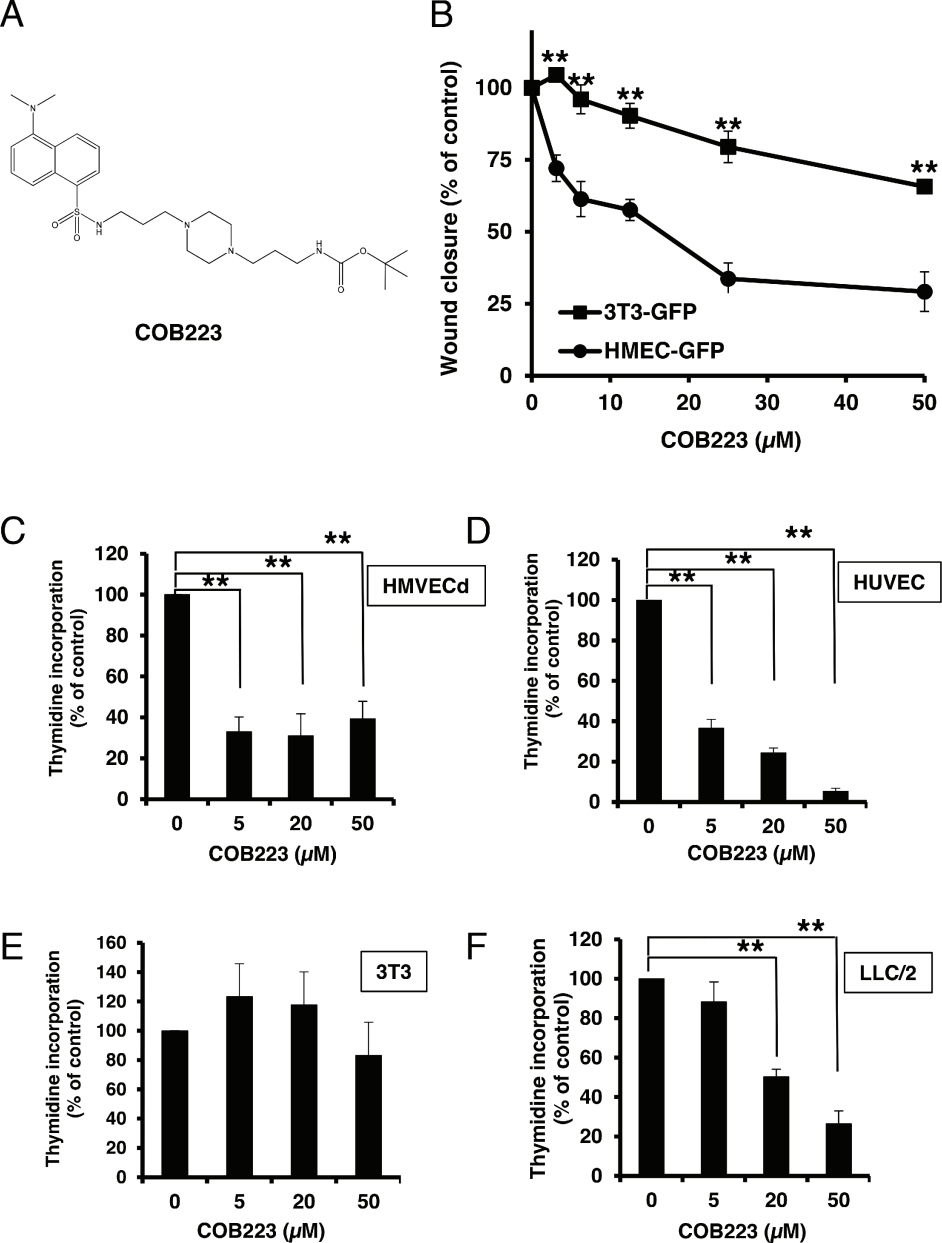
Effect of COB223 on cell migration and cell proliferation **(A)** Chemical structure of the leader compound COB223. **(B)** the dose-dependent effect of COB223 on cellular wound closure was measured as described in Material and Methods on either HMEC-GFP (circles) or 3T3-GFP (squares). **(C–F)** Cell proliferation was assayed by measuring ^3^H-thymidine incorporation into HMVECd (C), HUVEC (D), 3T3 (E) or LLC/2 (F) cell cultures as described in Material and Methods. The results are expressed as means +/− SEM from 3 independent experiments. ** : *p* < 0.01

Using this assay, we screened the academic library of the University of Grenoble, which is composed of 1360 original molecules. Setting the threshold of significant responses at 75% of the maximal inhibition, we identified 80 inhibitory molecules. At this stage, we reasoned that molecules that would affect the cytoskeleton dynamics or the adhesion properties of any cell type would be positive in this assay but would not represent good candidates for further development. We thus decided to perform a secondary counterscreen of the first 80 selected compounds on GFP-expressing 3T3 fibroblasts (3T3-GFP) using the same scratch assay. We then focused our interest on the 5 molecules that inhibited more strongly HMEC-GFP than 3T3-GFP wound healing. Among the most active molecules were several polyamine derivatives. We synthesized a series of 30 analogs and tested them at various concentrations both on HMEC-GFP and 3T3-GFP cells. The IC50 of these molecules for wound healing inhibition on both cell types is presented in Table 1. The most potent compound on HMEC-GFP was COB223 with an IC50 of 5 μM (Figure 2A, 2B). COB223 was 5 times less efficient at inhibiting wound closure of 3T3-GFP cells (Figure 2B). COB227 was also an interesting compound as, although its IC50 on HMEC-GFP (18 μM) was larger than that of COB223, its specificity for endothelial over fibroblastic cells was better (Ratio of IC50s = 14). The structure-activity relationship analysis indicated that aliphatic polyamines (ethylenediamine, diaminopropane, putrescine) or dansyl-cadaverine were inactive on both cell types. Careful analysis of the efficacy of the series of 30 analogs indicated that inhibition requires the presence of two hydrophobic substituents linked through amino/amido-containing chains. Dansyl and naphthalimide aromatic groups, found in COB223 and COB227 respectively, appeared very efficient. The presence of the diaminomethyl group on dansyl is critical for the activity of COB223 as shown by the lower efficiency of COB235 (IC_50_ is 8 times higher than that of COB223). COB223 and COB227 contain a tert-butyloxycarbonyl function (BOC group) as a second hydrophobic group. Replacement of BOC by an amino group decreased the efficiency and the specificity (compare 05-102-L-B09 vs 05-06-L-D03, or COB227 vs COB228). We also tested the effect of two dansyl groups (05-06-L-F11, COB222, COB295). The molecules showed interesting properties but the lack of water solubility precluded further development. Replacing the amine-containing linker of COB223 by a diethylene glycol chain in COB296 did not modify the activity, thus confirming the major importance of the dansyl and BOC groups whereas the linking chain may be of various length and chemical nature.

**Table 1:** Structure and activity of COB223 and its chemical analogs on HMEC-GFP and 3T3-GFP wound closure Each compound was tested at various concentrations (3.125 μM to 100 μM) in the presence of 10% FCS in cell scratching assays using either HMEC-GFP or 3T3-GFP cells. Quantitation of wound closure after 24 h allowed to determine the IC50 of the compound for each cell type. The forelast column indicates the ratio between the IC50s on both cell types. The last column indicates logP for each structure.

Compound	Formula	IC50 HMEC-GFP	IC50 3T3-GFP	Activity(HMEC vs 3T3)	logP
COB223	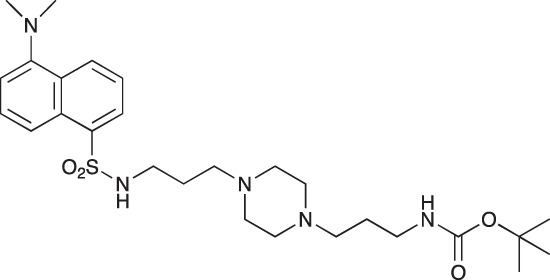	5	25	5	2.6
COB295	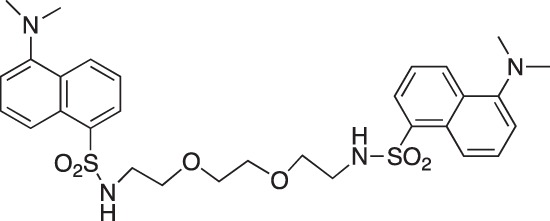	5	ND		3.5
COB296	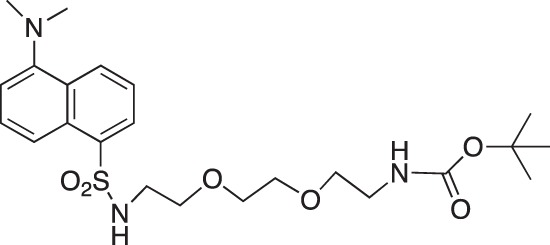	5	ND		2.5
COB275	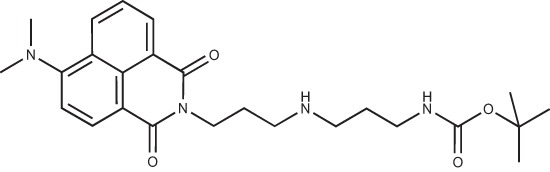	12.5	50	4	2.5
COB278	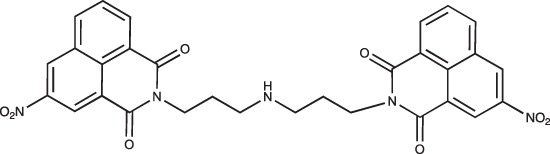	12.5	25	2	3.2
05-06-F11	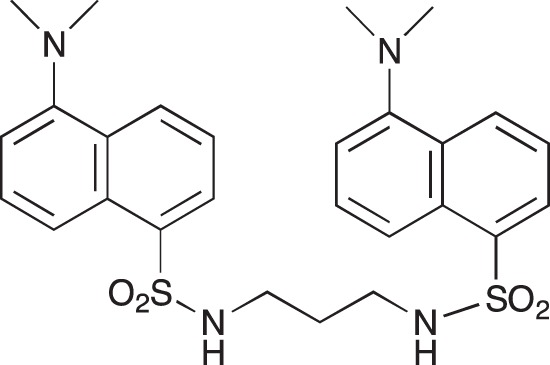	15	40	2.6	3.7
COB227	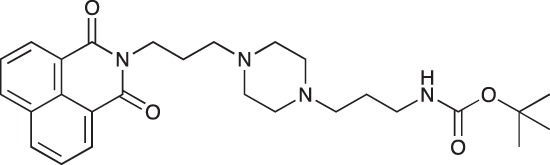	18	250	13.9	2.6
05-102-B09	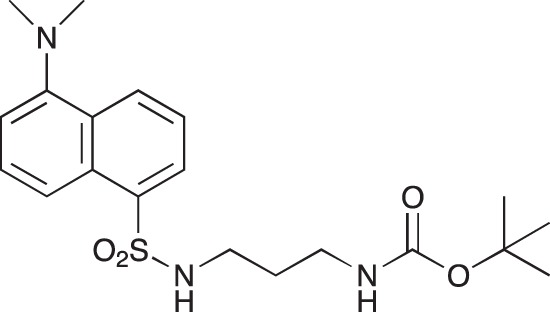	18	150	8.3	2.6
COB238	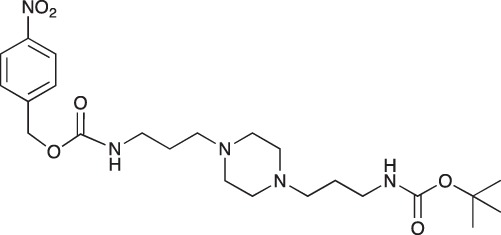	20	> 50	> 2.5	0.9
COB221	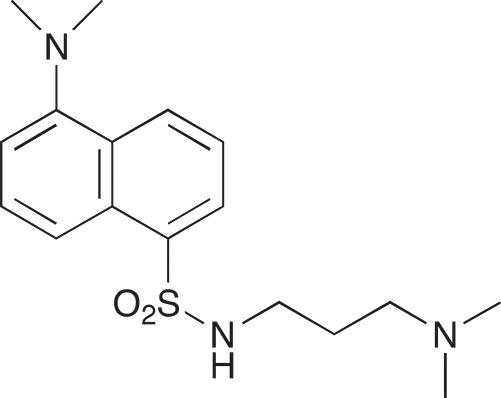	20	150	7.5	1.9
COB228	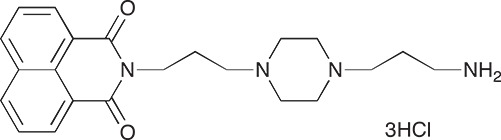	25	40	1.6	1.1
COB281	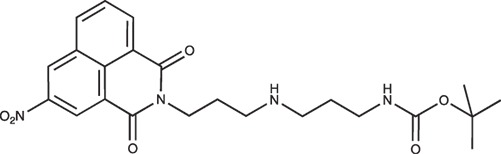	25	> 50		2.3
COB280	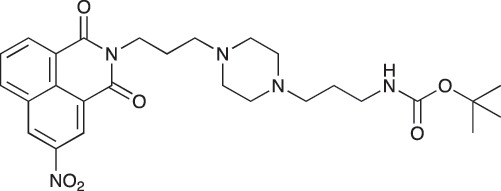	25	> 100		2.5
05-06-L-D03	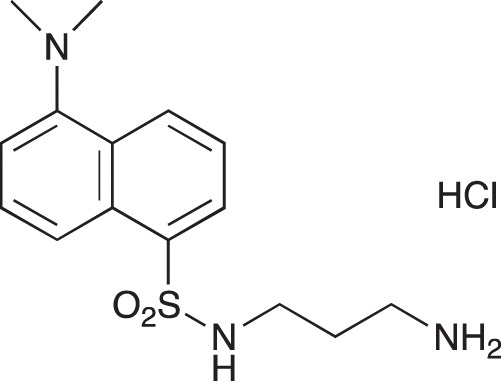	25	200	8	1.2
W7	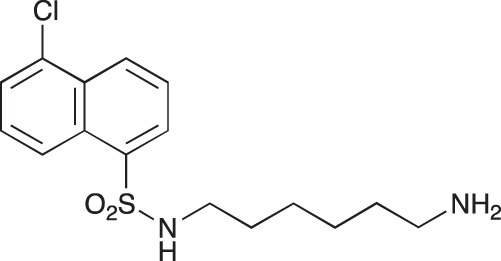	35	35	1	3.0
COB235	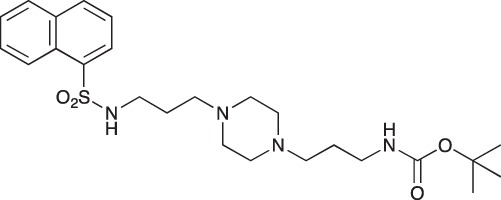	40	> 50	> 1.25	2.4
COB220	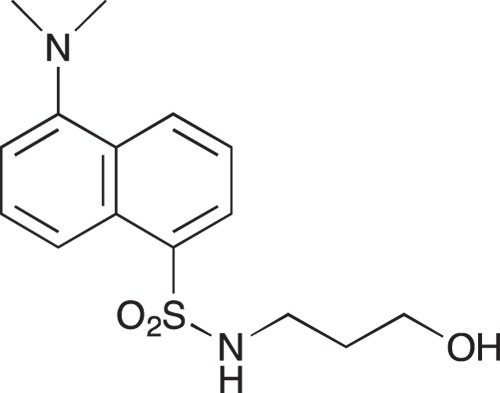	40	> 50	> 1.25	1.2
COB236	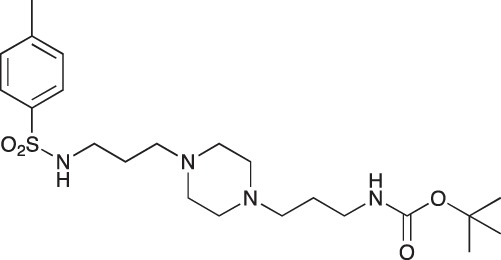	> 50	> 50		2.0
COB237	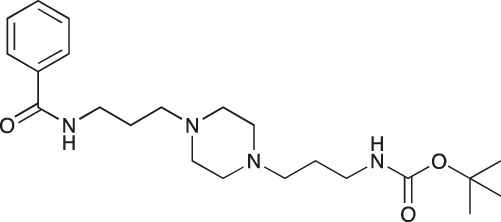	> 50	> 50		1.7
COB224	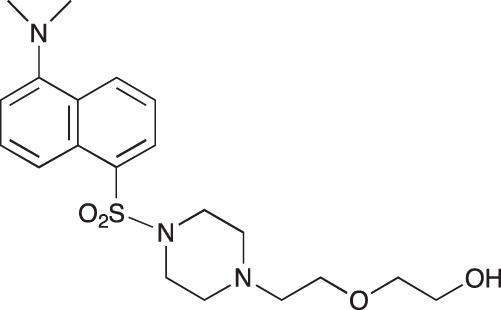	> 50	> 50		1.3
COB225	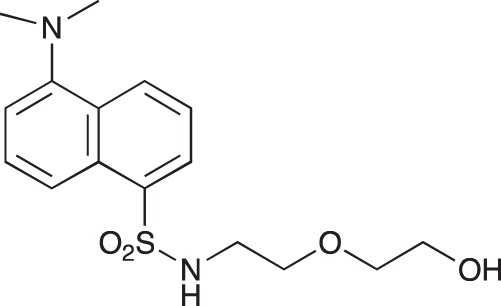	> 50	> 50		1.1
COB226	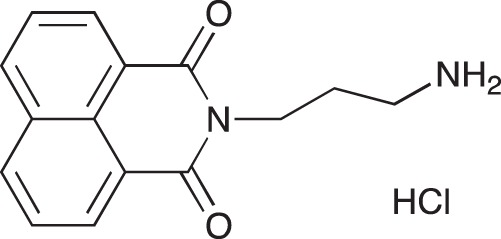	> 50	> 50		1.1
COB222	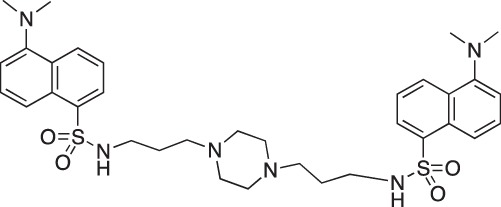	> 50	> 50		3.6
dansylcadaverine	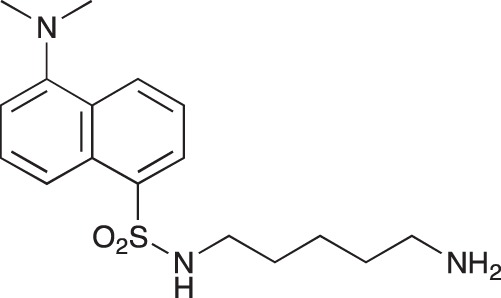	> 50	> 50		2.1
COB274	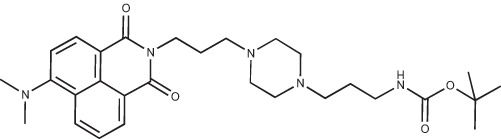	> 100	ND		2.7
COB276	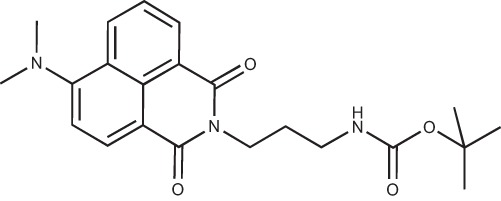	> 100	ND		2.7
COB277	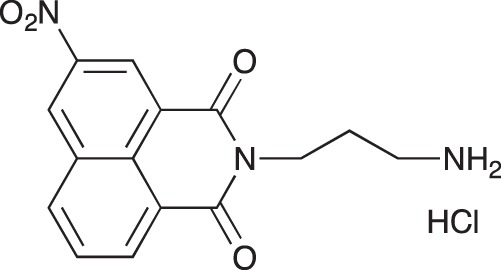	> 100	ND		2.6
COB279	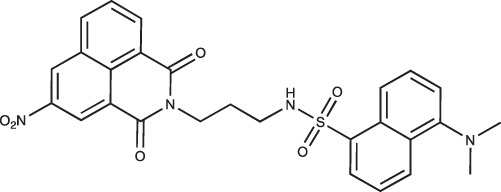	> 100	ND		3.6
COB282	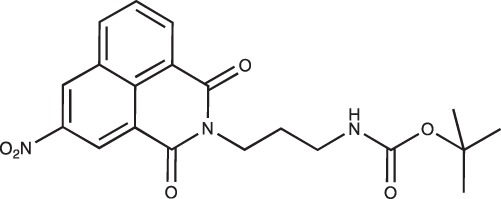	> 100	ND		2.6
COB283	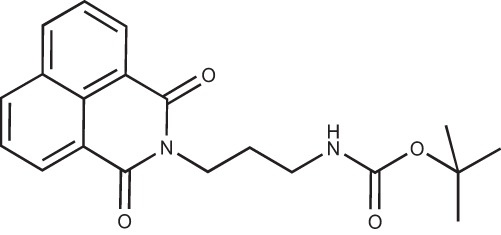	> 100	ND		2.6
Ethylene diamine	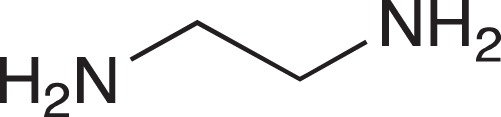	> 100	ND		
1,3-diaminopropane	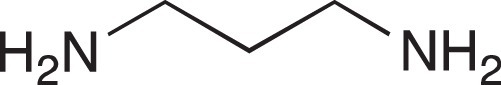	> 100	ND		
putrescine	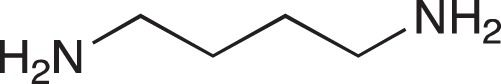	> 100	ND		

**Figure 3:**
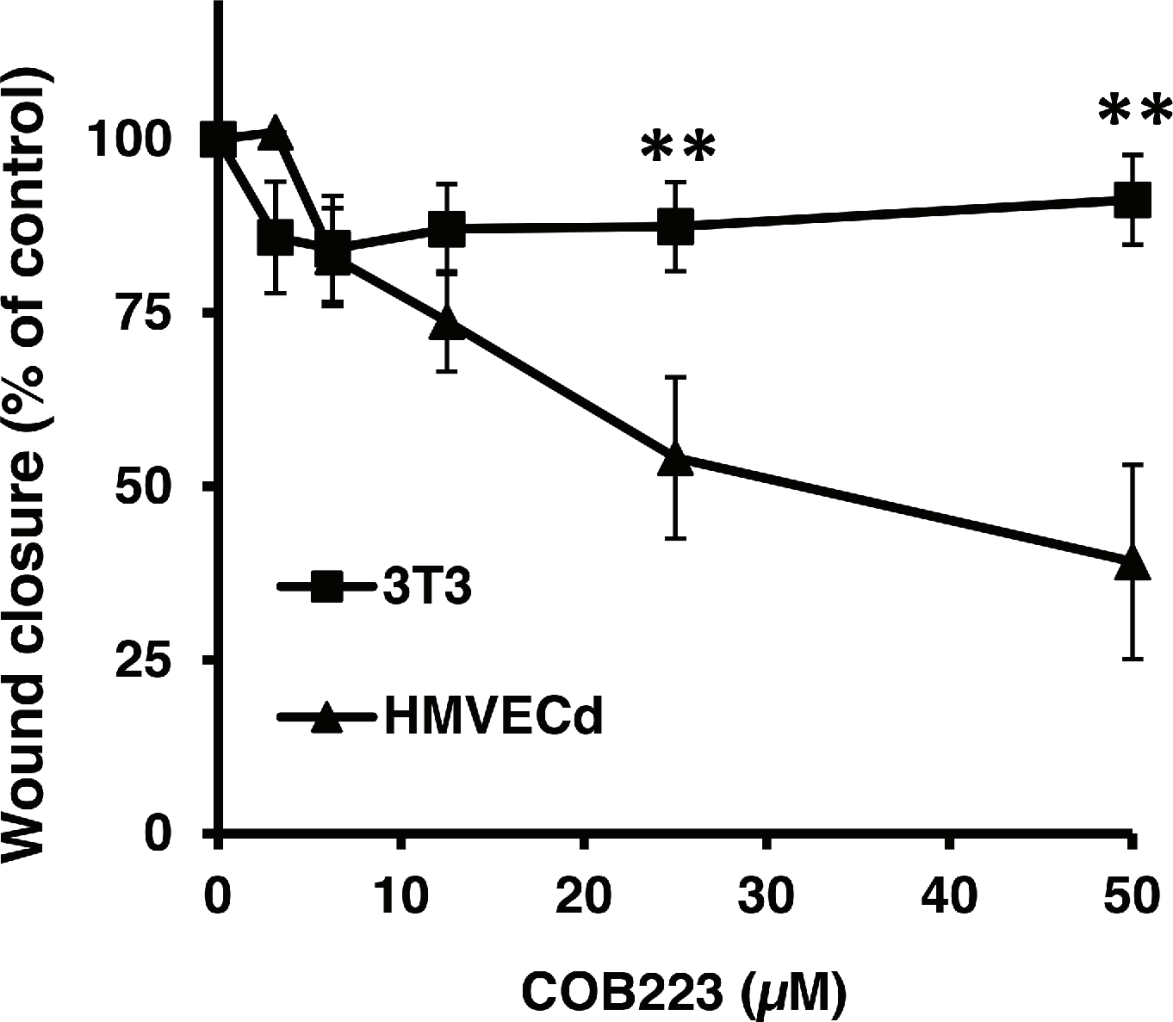
Endothelial cell-specific inhibition of wound closure by COB223 The dose-dependent effect of COB223 on cellular wound closure was measured as described in Material and Methods on either HMVECd or on 3T3 cells. The results are expressed as the percentage of wound closure (with closure observed in the presence of 10% FCS taken as 100%) : mean values +/− SD from three experiments.

As the screening was performed with GFP-expressing cell lines that might behave differently from primary endothelial cells, we measured the dose-dependent effect of COB223 on primary dermal microvascular endothelial cells (HMVECd) and on 3T3 fibroblasts (Figure 3). COB223 appeared to be less active on HMVECd (EC50 = 25 μM) than on HMEC-GFP (EC50 = 5 μM) but its preferential action on endothelial cells versus fibroblasts was still observed.

As wound healing in the scratch has been shown to result from activation of both cell migration and cell proliferation, we repeated this experiment in the presence of mitomycin C, a potent cell proliferation inhibitor. As shown in Figure S1A, wound healing was slowed down in the presence of mitomycin C confirming the implication of cell proliferation to the healing process, but COB 223 still inhibited endothelial cell wound closure in the absence of any cell proliferation. To confirm this effect on endothelial cell migration, we also used the Boyden chamber assay. As shown in Figure S1B, the migration of human microvascular endothelial cells towards a chemo-attractant (fetal calf serum) was significantly inhibited by COB223. These two experiments clearly indicate that endothelial cell migration, and probably also endothelial cell proliferation, are inhibited by COB223.

### Anti-proliferative activity of COB223

We then checked the effects of the leader compound COB223 on cell growth. As shown in Figure 2C and 2D, COB223 inhibited thymidine incorporation into both HMVECd and HUVEC endothelial cells at concentrations above 5 μM. We also checked the effects of COB223 on 3T3 fibroblast and LLC/2 lung carcinoma cell proliferation (Figure 2E and 2F). Interestingly, COB223 appeared to have no effect on 3T3 cells (as already observed on cell migration) but inhibited LLC/2 cell proliferation although less efficiently than it did on endothelial cells. These effects were confirmed by cell cycle analysis using a FACS cytometer (data not shown) and by measuring *in vitro* growth curves by cell counting (Figure S2).

**Figure 4:**
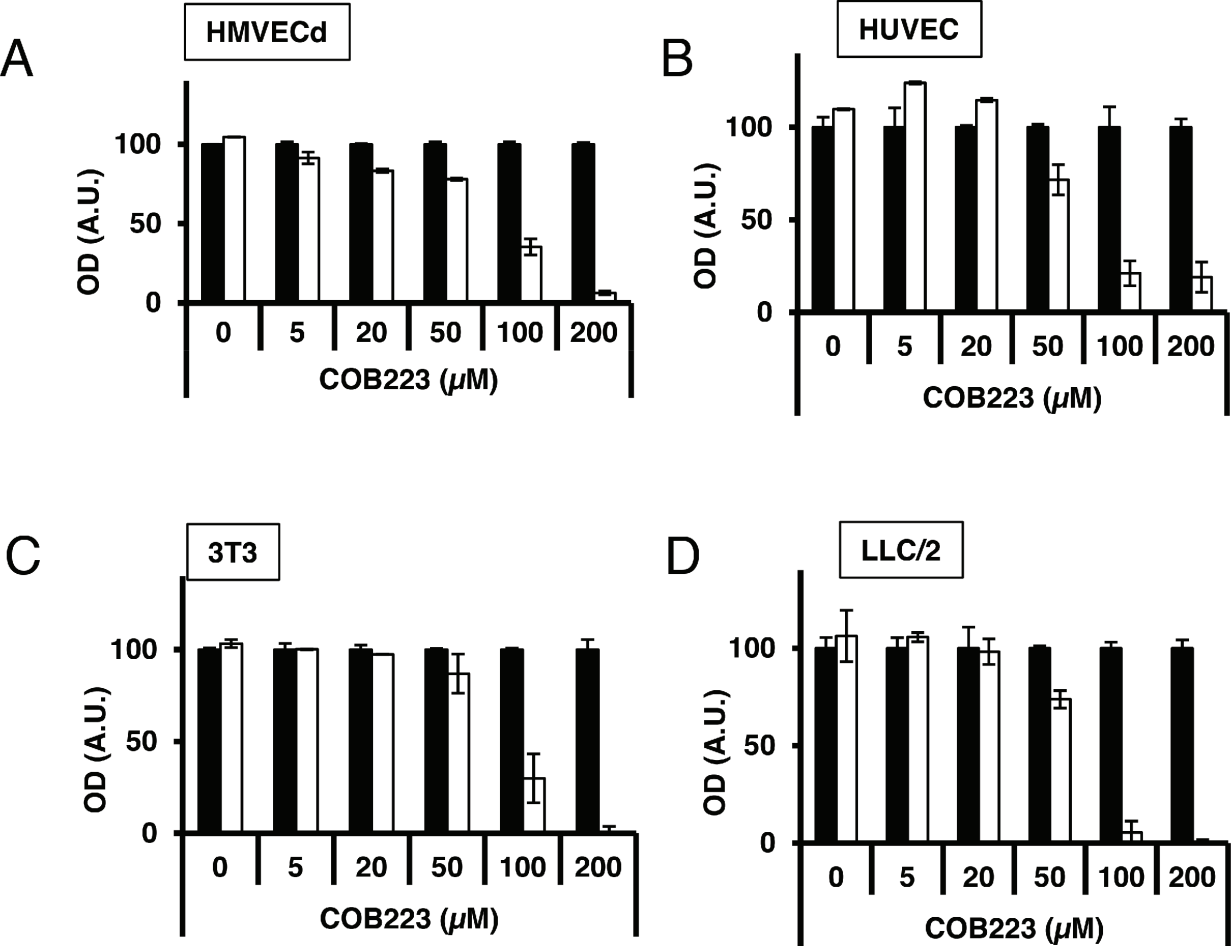
Cytotoxicity of COB223 Cellular toxicity was determined on confluent HMVECd **(A)**, HUVEC **(B)**, 3T3 **(C)** or LLC/2 **(D)** cell cultures kept in minimal serum conditions and treated by various concentrations of COB223 (white bars) or vehicle (black bars) for 24 h. The number of viable cells was quantitated using the tetrazolium blue assay, as described in Material and Methods. The results are expressed as means +/− SEM from 3 independent experiments. * : *p* < 0.05 ; ** : *p* < 0.01

In parallel, we evaluated the cytotoxicity of COB223 on these four cell types by incubating confluent cells with various concentrations of this compound for 24 h at 37°C (Figure 4). It clearly appeared that COB223 was cytotoxic on all cell types at concentrations larger than 50 μM. The major effects observed on endothelial cell proliferation and migration at 5 to 20 μM are thus not attributable to any cytotoxic effect.

### Confirmation of the anti-angiogenic activity of COB223 *in vitro*

**Figure 5:**
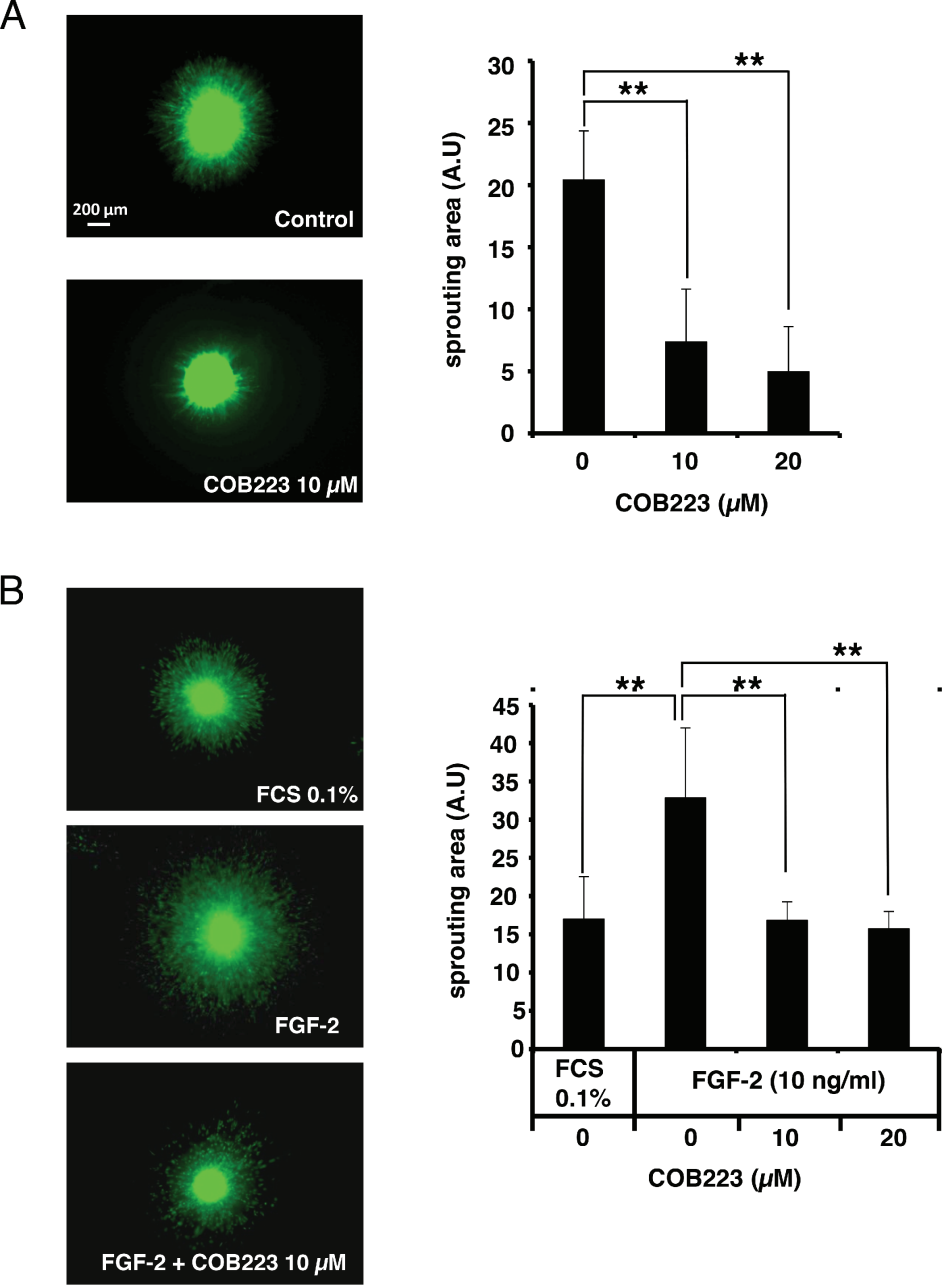
Effect of COB223 on sprouting of HMEC-GFP spheroids into collagen gels **(A)** Spheroids of HMEC-GFP cells were grown in collagen gels containing the indicated concentrations of COB223. The sprouting area was measured from images taken at day 4 using Cell Profiler software. **(B)** Spheroids of HMEC-GFP cells were grown in collagen gels containing either 0.1% FCS or 10 ng/ml FGF-2 and the indicated concentrations of COB223. The sprouting area was measured from images taken at day 3 using Cell Profiler software. Data are the mean +/− SD from the analyses of 10–12 spheroids (**p < 0.01). Each graph is representative of at least 4 independent experiments.

We then wished to establish that COB223 was still active in more biologically relevant *in vitro* angiogenesis assays, in particular in 3-dimensional assays that mimic endothelial sprouting in the extracellular matrix. We grew HMEC-GFP spheroids in collagen I gels in the absence or the presence of 10 ng/ml FGF-2 and either 10 or 20 μM COB223 and we observed the spontaneous radial sprouting of endothelial cells after 96 h of culture. As shown in Figure 5A and 5B, 10 μM COB223 inhibited both basal and FGF-2-stimulated sprouting by 50% and 70%, respectively.

**Figure 6:**
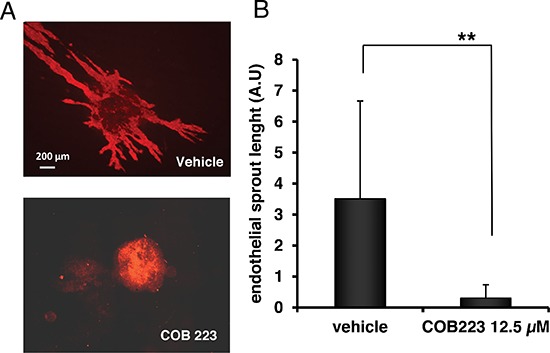
Effect of COB223 on vascular sprouting of embryoid bodies into collagen gels **(A)** Embryoid bodies derived from mouse embryonic stem cells (as described in Material and Methods) were grown in collagen gels in the presence of 12.5 μM COB223 or vehicle. On day 10 of culture, cells commited into the endothelial lineage were visualized by *in toto* immunofluorescent staining using anti-CD31 antibodies. **(B)** The total surface occupied by endothelial sprouts was measured by image analysis of the pictures taken under an epifluorescence microscope. Data are means +/− SD of analyses performed on 17 and 19 spheroids per condition. Similar results were obtained in 2 independent experiments. ** : *p* < 0.01.

We then used a second angiogenesis assay in which embryonic stem (ES) cells are allowed to differentiate toward the endothelial lineage and to subsequently sprout from pre-formed spheroids into a collagen-I gel [8]. Endothelial sprouts can be visualized by CD31 immunostaining of *in toto* preparations (Figure 6). Adding COB223 (12.5 μM) to the collagen gel at day 6 clearly resulted in a complete inhibition of endothelial sprouting as shown in Figure 6A and 6B.

### COB223 inhibits angiogenesis *in vivo*

**Figure 7:**
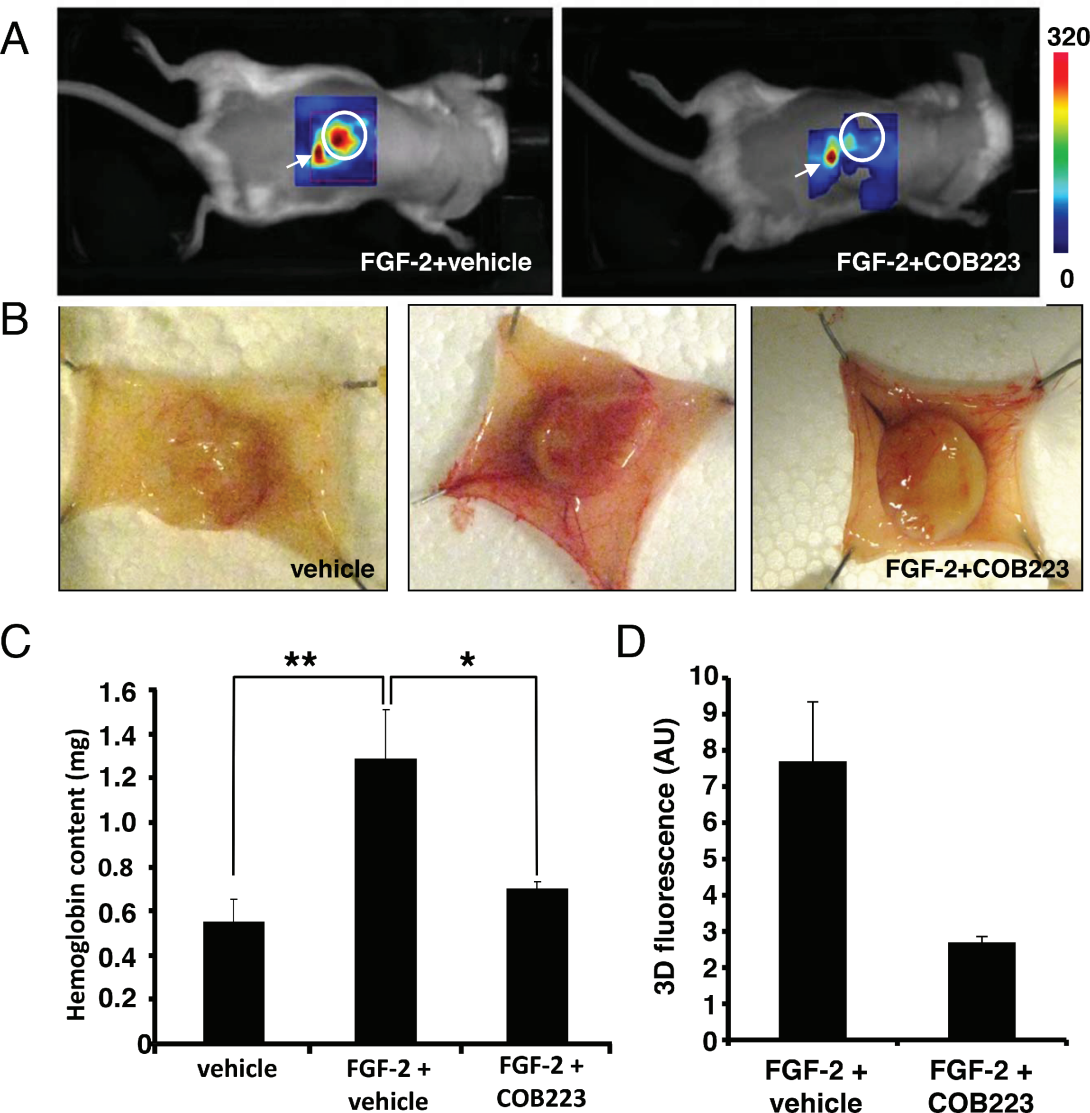
*In vivo* effects of COB223 on angiogenesis in the mouse sponge assay Cellulose sponges were implanted under the dorsal skin of Balb/c mice and injected several times with FGF-2 and either COB223 or its vehicle as described in Material and Methods. **(A)** At the end of the experiment (day 7), the neovasculature was visualized by non-invasive fluorescence imaging (see details in Material and Methods). **(B)** The mice were then sacrificed and the dorsal skin flaps were surgically collected and photographed. **(C)** The sponges were homogenized and their hemoglobin content was measured. **(D)** Intensity of fluorescence observed in the sponges in (A) In (A) and (D), 2 mice were analyzed in each group. In (B) and (C), 7 mice were analyzed in each group. * : *p* < 0.05 ; ** : *p* < 0.01

We then checked whether the anti-angiogenic effect of COB223 could be observed *in vivo*. We used the mouse sponge assay in which a cellulose sponge is soaked with FGF-2, a potent pro-angiogenic factor, and subsequently implanted under the dorsal skin of mice. FGF-2 is then injected every other day through the skin into the sponge in the presence or the absence of either COB223 or vehicle. After 7 days, the blood vessels and capillaries that have grown from the skin towards the surface of the sponge can be visualized by non-invasive image analysis using the fluorescent marker RAFT-(RGDc)_4_ (also known as Angiostamp^TM^), as recently established [9]. After imaging, the sponges can be recovered from sacrificed mice, photographed and the angiogenic response can be monitored by determination of the hemoglobin content of the homogenized sponges. As shown in Figure 7, both techniques conclusively showed a strong inhibition of the neovascular response to FGF-2 in the presence of COB223.

### COB223 reduces tumor growth and microvessel formation

**Figure 8:**
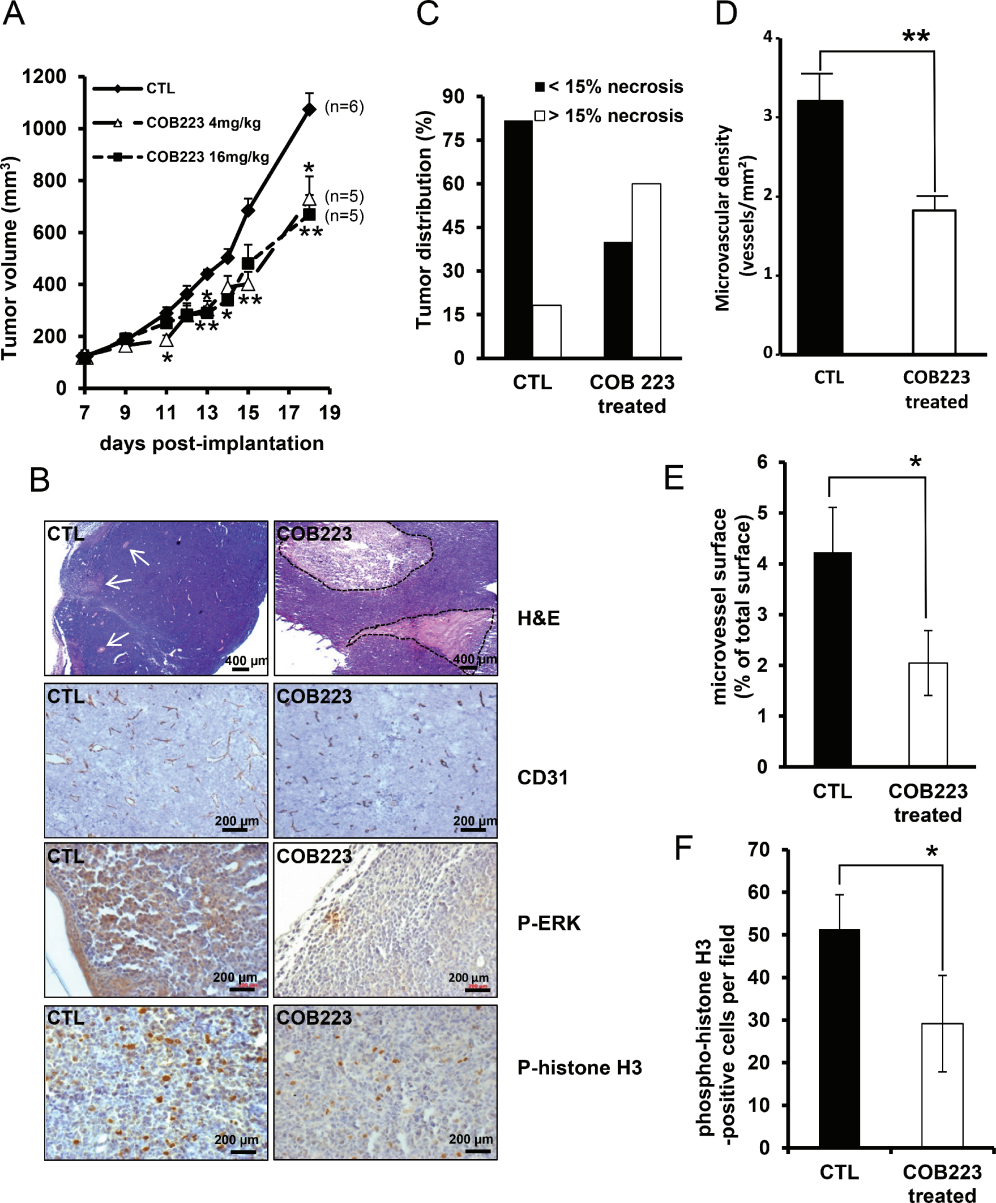
*In vivo* effects of COB223 on LLC/2 tumor formation **(A)** LLC/2 cells were injected under the skin of nude mice. Once the tumors had reached a size of 50 mm^3^, the mice were injected i.p. every other day either with 4 mg/kg or 16 mg/kg of COB223 or with vehicle alone. The volume of the tumors is plotted versus time. **(B)** Tumor sections from control and COB223-treated animals were stained with haematoxylin & eosin, revealing pale necrotic areas (indicated by white arrows or circled by dotted lines), and immunostained with anti-CD31 antibodies (blood vessels), anti-phospho-ERK1/2 and anti-phospho histone H3 (proliferation status). **(C)** 3 sections from each tumor were analyzed to determine which percentage of the tumors presented with more than 15% of their surface as necrotic. 10 control and 15 COB223-treated tumors were used in this analysis. **(D–E)** Image analysis of the CD31 immunostainings allowed to quantify microvessel density (D) and microvessel size (E). **(F)** Image analysis of the mean number of phospho-H3-positive nuclei per field in control and COB223-treated tumor sections.

Next, we used a murine xenograft tumor assay to determine the therapeutic potential of COB223. Nude mice were subcutaneously implanted with LLC/2 tumor cells and, once the tumor volume reached 50 mm^3^ (i.e. at day 7), were treated i.p. every other day either with 4 mg/kg or 16 mg/kg COB223 or with vehicle. Under COB223 treatment, we observed a similar decrease of tumor growth (32% to 38% reduction in tumor size at day 18 post-implantation) for both doses comparatively to mice treated with vehicle (Figure 8A). Although this inhibition is not drastic, it is interesting to note that the reduction in tumor size results from decreased growth rate since the slope of the growth curve is reduced under COB223 treatment. Hematoxylin/eosin staining of COB223-treated tumor sections revealed enlarged necrotic areas as compared to untreated tumors (Figure 8B). Quantification of this necrotic process indicated that 20% of untreated tumors versus 60% of COB223-treated tumors presented necrotic areas larger than 15% of their surface (Figure 8C). In addition, we investigated tumor vascularization by CD31 immunostaining (Figure 8B). A significant decrease in the density and the size (luminal surface) of the microvessels was observed in the non-necrotic part of COB223-treated tumors, as quantified in Figure 8D and 8E. This allowed us to speculate that the anti-tumoral effect of COB223 might be caused, at least in part, by its anti-angiogenic action. However, as we observed that COB223 reduces also the proliferation of tumor cells, we analyzed the proliferative status of control and COB223-treated tumors using two markers of cell division, phospho-ERK1/2 and phospho-histone H3 (Figure 8B). The number of positive cells for these two markers was significantly decreased in sections from COB223-treated tumors (Figure 8F).

As COB227 showed a better ratio of *in vitro* activity between endothelial cells and fibroblasts, we also checked its effects on *in vivo* LLC/2 tumor growth. We observed no inhibition of tumor growth and therefore stopped the development of this series of naphtalimide compounds, as this lack of effect probably reflected poor pharmacodynamic or pharmacokinetic properties.

### Identification of COB223 mechanism of action

**Figure 9:**
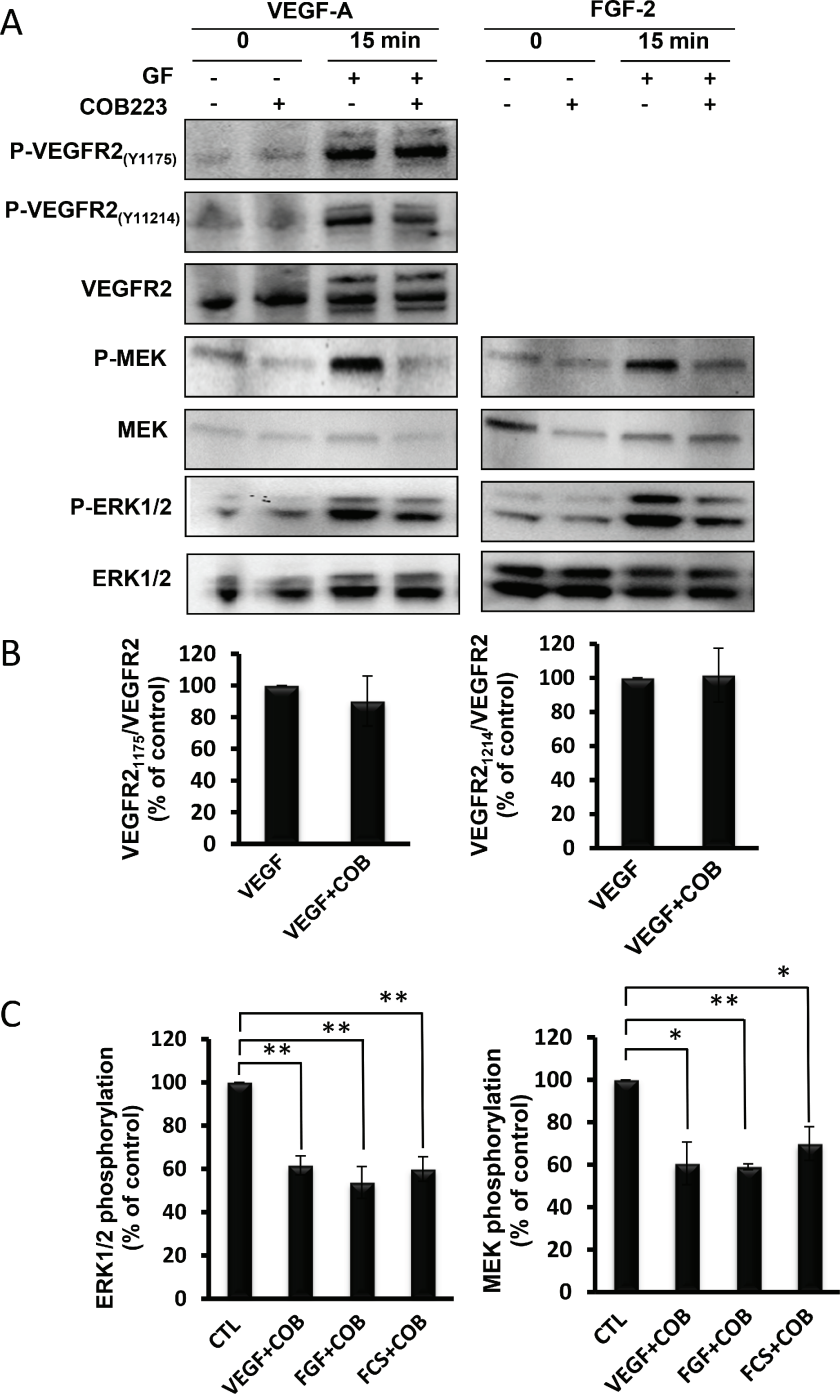
Effect of COB223 on growth factor-induced VEGF-R2, MEK and ERK phosphorylations **(A)** Serum-starved HUVEC cells were incubated for 15 min in the presence of either 10 ng/mL VEGF-A or 20 ng/mL FGF-2 and in the absence or the presence of 25 μM COB223. The phosphorylations of VEGFR2 on tyrosine 1175 or tyrosine 1214, of MEK and ERK1/2 were then analyzed by Western blot analysis of the cell lysates as described in Material and Methods. Western blots for total VEGFR2, MEK and ERK1/2 were used as loading controls. The blots shown in Figure 9A were obtained in one representative experiment. **(B)** The hybridization signals observed in 5 similar but independent experiments were quantitated and the ratios between phospho-VEGFR2(Y1175) or (Y1214) and VEGFR2 signals were normalized to the value observed in the absence of COB223. **(C)** The hybridization signals observed in 5 similar but independent experiments were quantitated and the ratios between phospho-ERK1/2 and ERK1/2 and between phospho-MEK and MEK were normalized to the values observed in the absence of COB223. Additional experiments performed with 10% fetal calf serum (FCS) as a mitogen were included in this analysis. * : *p* < 0.05 ; ** : *p* < 0.01

We first wondered whether COB223 required to be internalized to exert its biological effect. For this, we synthesized a covalent complex between BSA and COB223 and purified it. Whereas COB223 (which can be visualized by its fluorescent dansyl group) was rapidly taken up and internalized in endothelial cells (Figure S3), COB223-BSA did not appear to enter these cells (data not shown). Interestingly, COB223-BSA was also unable to inhibit wound closure in endothelial cells, indicating that its mode of action is intracellular. In agreement with this conclusion, we observed that binding of ^125^I-VEGF-A_121_ to HUVEC cells was unaffected by the presence of COB223 whereas it was efficiently displaced by unlabeled VEGF (data not shown). We then evaluated whether COB223 could act as a protein-kinase inhibitor, although its structure does not resemble those of ATP-competing inhibitors such as sorafenib or sunitinib. *In vitro* kinase assays were performed with a panel of purified protein-kinases involved in mitogenic signaling, including VEGFR2, FGFR1, FGFR2, PDGFRß, Met, Raf, MEK1, ERK1, ERK2, PKCα, PKCβ_I_, PKCβ_II_, PKCζ as indicated in Table 2. None of them was inhibited by COB223 at concentrations up to 50 μM. We then analyzed the effect of COB223 on VEGF-induced phosphorylations of VEGFR2, MEK and ERK1/2 (Figure 9). COB223 did not alter the autophosphorylation of VEGFR2 on tyrosine residues 1175 (this site mediates activation of the ERK pathway) and 1214 (this site mediates the activation of the p38 MAP kinase pathway) [10] (Figure 9A, 9B). In contrast, VEGF-induced phosphorylations of MEK and ERK1/2 were significantly inhibited in the presence of COB223. When FGF-2 or fetal calf serum were used as mitogenic activators of the MAP kinase pathway, we observed a similar inhibitory effect of COB223 on MEK and ERK1/2 (Figure 9A, 9C). A detailed dose-response study indicated that inhibition of MAP kinase phosphorylation was detectable at a concentration of 5 μM and was statistically significant at 20 μM. This inhibition also appeared to be somehow cell-specific since COB223 did not affect FGF2-induced or serum-induced phosphorylation of ERK1/2 in 3T3 fibroblasts (data not shown). It was also specific of the p42/p44 MAP kinase pathway as activation of p38 MAP kinase phosphorylation in HUVEC cells by VEGF or serum was unaffected by COB223 (data not shown). The activation of src phosphorylation by VEGF in HUVECs was also unaffected by COB223 (data not shown).

**Table 2:** List of protein-kinases that are unsensitive to COB223 *in vitro* *In vitro* kinase assays were performed with each of these purified protein-kinases in the presence or the absence of various concentrations (0.5 up to 100 μM) of COB223. None of these kinase activities was inhibited by more than 5% in the presence of these concentrations of COB223.

**Receptors:** EGFR, FGFR1, FGFR2, VEGFR2 (KDR), Insulin Receptor, PDGFRß, TGFßR1, Met, Tie2
**Intracellular protein-kinases:** MEK1, ERK1, ERK2, Raf, FAK, CK2, CDK2/cyclin A, CDK2/cyclin E, CDK6/cyclin D3, PKCα, PKCβI, PKCβII, PKC ζ.

**Figure 10:**
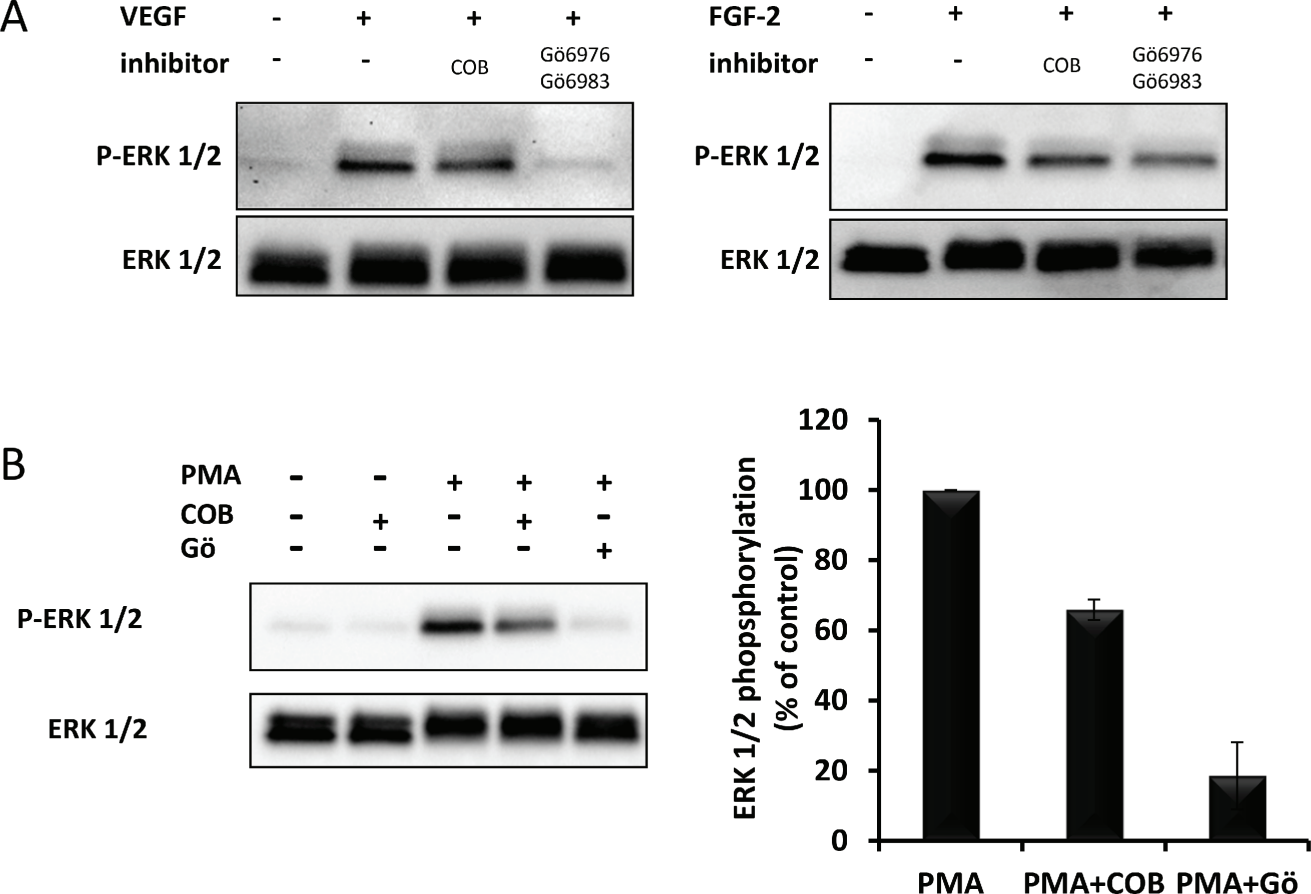
Effect of PKC inhibitors on PMA-, VEGF- and FGF2-induced ERK phoshorylation **(A)** Serum-starved HUVEC cells were incubated for 15 min in the presence of 10 ng/mL VEGF-A or 20 ng/mL FGF-2 and in the absence or the presence of 25 μM COB223 or 5 μM Gö6983 and 5 μM Gö6976. The phosphorylation ERK1/2 was then analyzed by Western blot analysis of the cell lysates as described in Material and Methods. Western blots for total ERK1/2 were used as loading controls. **(B)** Serum-starved HUVEC cells were incubated for 5 min in the presence of 200 nM of PMA and in the absence or the presence of 25 μM COB223 or 5 μM Gö6983 and 5 μM Gö6976. The hybridization signals observed were quantitated and the ratio between phospho-ERK1/2 and ERK1/2 was normalized to the values observed in the absence of COB223. Similar results were obtained in 2 independent experiments.

**Figure 11:**
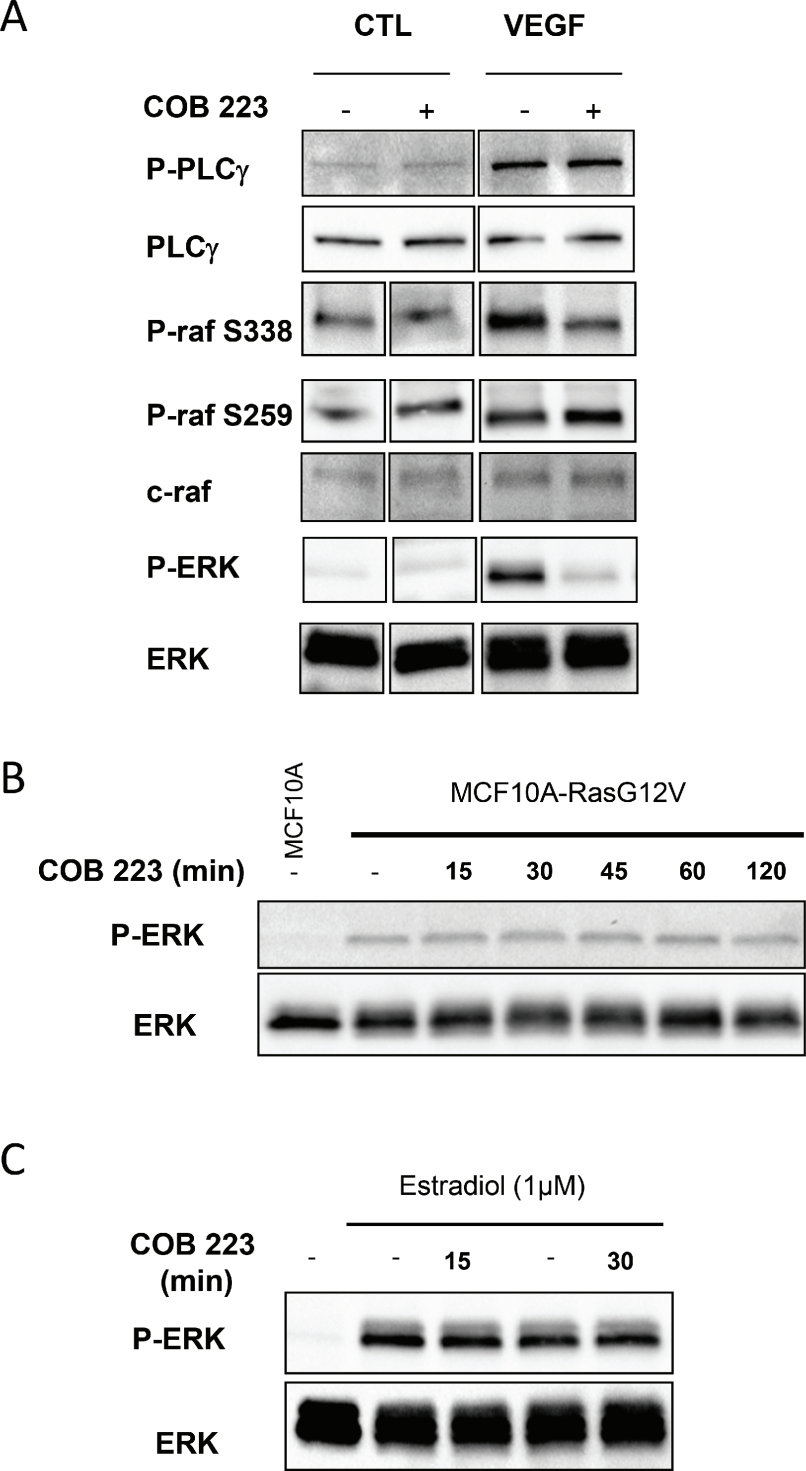
Effect of COB223 on mitogenic or oncogenic activation of the Ras /MEK/ERK signaling pathway **(A)** Serum-starved HUVEC cells were incubated for 15 min in the presence of 10 ng/mL VEGF-A and in the absence or the presence of 25 μM COB223. The phosphorylations of PLCγ on tyrosine 783, of Raf on serine 338 or on serine 259 and of ERK1/2 were then analyzed by Western blot analysis of the cell lysates as described in Material and Methods. Western blots for total PLCγ, Raf and ERK1/2 were used as loading controls. **(B)** MCF10A cells overexpressing an oncogenic version of Ras (MCF10A-RasG12V) were treated for the indicated periods of time by 25 μM COB223. Total ERK1/2 and phospho-ERK1/2 were then visualized by Western blotting of the cell extracts. Non-treated regular MCF10A cells were analyzed as a control (first lane). **(C)** CCL-39 fibroblasts expressing a constitutively active Raf in an estrogen-responsive manner were stimulated for 3 h with estradiol and treated for 15 or 30 min with 25 μM COB223. Total ERK1/2 and phospho-ERK1/2 were then visualized by Western blotting of the cell extracts. Non-treated cells were analyzed as a control (left lanes).

One important step between the phosphorylation of growth factor receptors and the activation of ERK1/2 is the activation of Ras. Unlike most other receptor tyrosine kinases, VEGFR2 activates Ras, not via GRB2-SOS, but via phospho-Y1175-dependent phosphorylation of PLCγ and subsequent activation of protein kinases-C (PKCs) [11, 12]. Although the exact mechanism of VEGFR2-induced Ras activation is still unclear, one proposed mechanism involves PKC-mediated activation of sphingosine kinase-1 (SPK1) [13]. In agreement with these statements, we observed that chemical inhibitors of PKC (Gö6976+Gö6983), completely inhibited VEGF-induced ERK1/2 phosphorylation in HUVECs whereas they only partially inhibited FGF2-induced ERK1/2 phosphorylation (Figure 10A). In the same assay, COB223 inhibited the phosphorylation of ERK1/2 induced by the PKC activator PMA (Figure 10B), confirming that it is acting downstream of PKC. We then checked the phosphorylation of the upstream activator of MEK, namely Raf and of the downstream target of activated VEGFR2, namely PLCγ (Figure 11A). VEGF-induced tyrosine phosphorylation of PLCγ on residue Y783 was unaffected by COB223. In contrast, phosphorylation of Raf on residue S338 (the target of Ras activation) was inhibited in the presence of COB223 whereas Raf phosphorylation on residue S259 (a site reported as a direct target of PKA and Akt/PKB) was not. The same differential effect was observed on Raf phosphorylation when serum was used as a mitogenic stimulus (data not shown). We then attempted to measure the activation of Ras by using a pull-down assay in which the Ras-binding domain of Raf is used to catch and subsequently detect active Ras but we were unable to generate reproducible results. In order to circumvent this limitation, we checked the effect of COB223 on the phosphorylation of ERK1/2 induced by the constitutive expression of oncogenic Ras^G12V^ in MCF-10A cells (Figure 11B). COB223 did not modify this constitutive phosphorylation, suggesting that it is acting upstream of Ras. In agreement with this observation, COB223 did not inhibit ERK phosphorylation induced by estrogen in CCL-39 fibroblasts expressing a constitutively active Raf in an estrogen-responsive manner (Figure 11C).

## DISCUSSION

Anti-angiogenic drugs such as anti-VEGF antibodies (Bevacizumab) and tyrosine kinase inhibitors (Sorafenib, Sunitinib, Pazopanib, …) have proven some efficacy in the treatment of several metastatic cancers [14, 15]. Since their beneficial effect is only observed in association with chemotherapy, their true mechanism of action in the patients is still a matter of debate [16–18]. Moreover, the frequent appearance of resistance to these anti-angiogenic therapies have tempered the initial enthusiasm [19]. When resistance to a first line anti-angiogenic treatment emerges, changing the class of anti-angiogenic molecules (e.g. from Bevacizumab to a tyrosine kinase inhibitor) results in prolonged survival of the patients [20]. New anti-angiogenic molecules, acting through distinct mechanisms than the existing ones, are thus urgently required for second or third line treatments of angiogenic cancers.

In this work, we searched for such new anti-angiogenic molecules by screening an academic chemical library (from DCM, University Grenoble-Alpes, France) that contained original, never-patented molecules. We could identify a family of dansylated polyamines which showed marked cell specificity as they inhibited more strongly endothelial cell than 3T3 fibroblast migration and proliferation. The SAR study revealed that natural or synthetic aliphatic polyamines such as spermine, putrescine, diaminopropane or ethylenediamine were inactive and that both chemical goups (dansyl and BOC) present at each extremity of the leader compound COB223 were important. So far, no biological activity for such compounds (or closely structural analogs) has been described.

We wondered whether COB223 required to be internalized to exert its anti-angiogenic action. The fluorescent molecule COB223 is rapidly internalized by endothelial cells in a temperature-dependent manner. Interestingly, uptake of COB223 was less efficient in 3T3 fibroblasts (Figure S3) and appeared to correlate with the lesser biological activity of this compound on 3T3 cell proliferation and migration. In agreement with an intracellular site of action of COB223, we observed that coupling COB223 to BSA (BSA is not cell-permeable) suppressed its inhibitory effect on endothelial cell migration. In addition, we observed no competition of COB223 with VEGF-A for its cell surface receptors and no inhibition of VEGF-induced VEGFR2 tyrosine phosphorylation. Taken together, these observations support an intracellular mode of action for this molecule.

**Figure 12:**
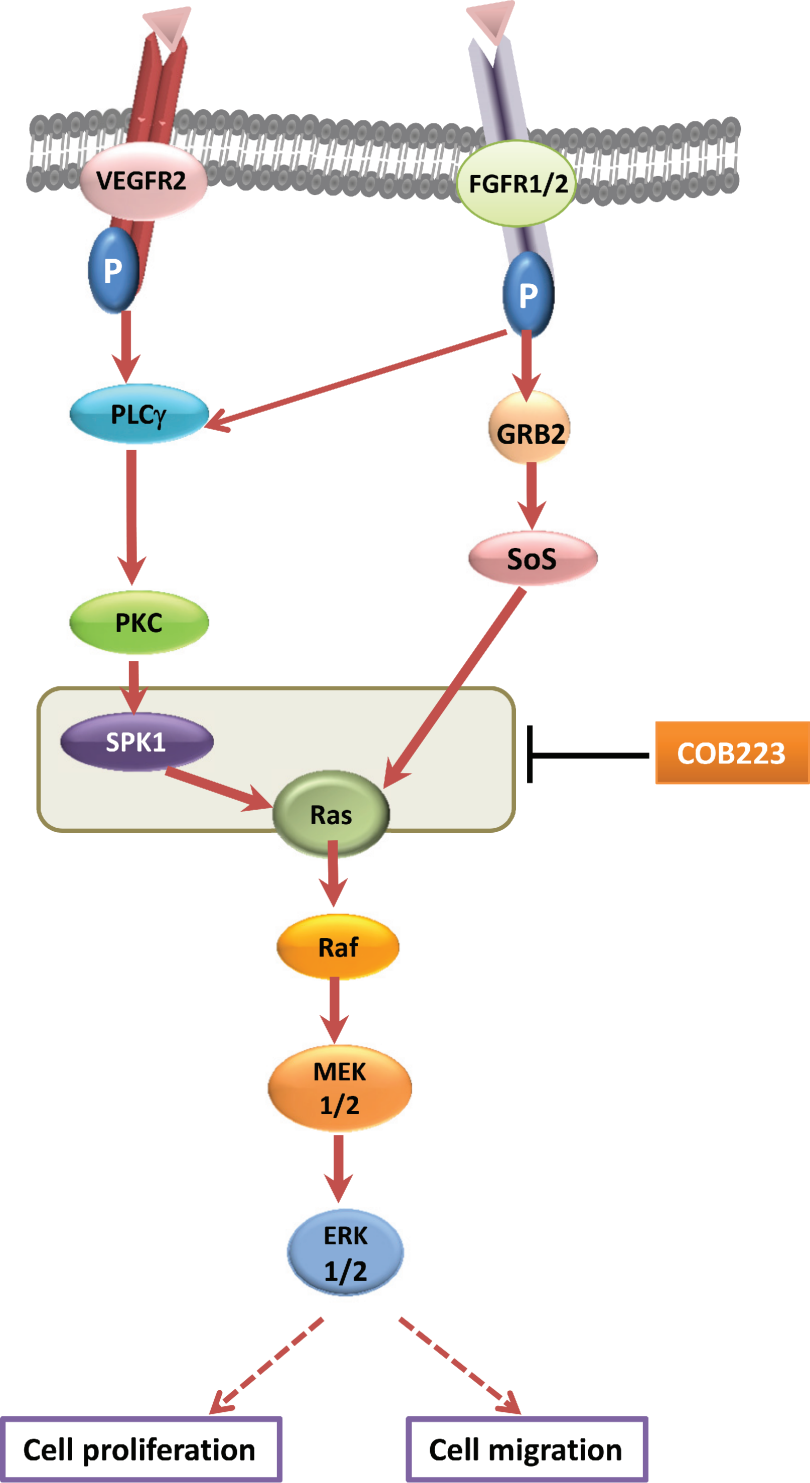
Mechanism of action of COB223 on the ERK pathway Full red arrows show the ERK signaling pathway that we established in HUVEC. The grey box indicates where the target of COB223 is located.

Careful and detailed analysis of the effects of COB223 on the signaling pathway activated in endothelial cells by VEGF and other endothelial mitogens such as FGF-2 and serum, led us to the conclusions that COB223 acts downstream of the activated growth factor receptors and upstream of activated Ras, Raf, MEK and ERK1/2 (Figure 12). Ras is a small G-protein acting downstream of growth factor receptors. In quiescent cells, Ras is bound to GDP and is inactive. Unlike most growth factors which activate Ras through the adaptor proteins Grb2 and the GDP/GTP exchange factor Sos, VEGF induces the recruitment of the adaptor protein TSAd (T cell-specific adaptor) and the effector protein PLCγ onto the phosphorylated residues Y951 and Y1175 of activated VEGFR2, respectively [12, 21]. Subsequent phosphorylation of PLCγ induces hydrolysis of the membrane phospholipid phosphatidylinositol (4,5)-bisphosphate into diacylglycerol and inositol 1,4,5-trisphosphate (IP3), which, in turn, activate the PKC isoforms PKCα, PKCβ and PKC ζ [22, 23]. One relevant substrate of VEGF-activated PKCs is sphingosine kinase 1 (SPK1), which converts sphingosine into sphingosine-1 phosphate (S1P). How S1P activates Ras is still an unanswered question but it was reported that this occurred through activation of GAPs (GTPase activating proteins) rather than through inhibition of GEFs (Guanine exchange factors) [13]. In its GTP-bound form, Ras recruits the protein kinase Raf at the plasma membrane and subsequently activates MEK and ERK1/2 (p42/p44 MAPK). As we observed that COB223 inhibits not only endothelial cell migration but also cell proliferation, the inhibition of Ras activation would certainly be decisive for triggering cell proliferation arrest. It was more surprising to us that COB223 did not affect the activation of p38 MAPK as this pathway, which is also stimulated by activated VEGF receptors, has been reported to trigger cell migration through phosphorylation of heat-shock protein 27 [24] and LIM kinase 1 [25]. One possible explanation is that other signaling pathways implicated in cell migration such as Rho-A or Focal Adhesion Kinase (FAK) are affected downstream of Ras inhibition [26]. More detailed studies will be required to elucidate these mechanisms.

Intraperitoneal administration of COB223 to LLC/2 tumor-bearing mice resulted in a significant decrease the rate of tumor growth, suggesting a direct anti-proliferative or pro-apoptotic effect. No difference was observed between the two doses tested, indicating that 4 mg/kg already produced a maximal effect. This is a much lower dose than those routinely used with other chemical angiogenesis inhibitors in similar models: 30–60 mg/kg for sorafenib or 50 mg/kg for sunitinib. Although the compound is not yet optimized, this suggests that its bioavailability is excellent. The intensity of fluorescence of the dansyl group of COB223 was unfortunately not sufficient to allow a detailed study of the biodistribution of the molecule and would need further addition of a radioactive or a more highly fluorescent tag. The tolerance of the molecule appeared excellent as no adverse effect was observed over a two week-long treatment with up to 16 mg/kg COB223. The major effects observed in tumors from COB223-treated mice were a decrease in the density and the size of the tumoral microvessels and a marked increase in necrosis. As we showed that COB223 is also cytostatic for LLC/2 tumor cells *in vitro*, the observed antitumoral effects might result from both an anti-angiogenic effect and a direct anti-proliferative effect on tumor cells. COB223 appears to inhibit the growth factor-induced Raf/MEK/ERK protein kinase cascade but to be unable to inhibit the activation of these kinases by an oncogenic version of Ras. It should therefore be efficient on tumor models bearing an activated Ras pathway (due e.g. to growth factor receptor overexpression) but inactive on those presenting oncogenic Ras mutations. It is worth noting that, in Lewis lung carcinoma, Ras is not mutated but is activated due to lack of p66Shc expression [27].

Several targeted therapies that inhibit the Ras-Raf-MEK-ERK pathway have been developed by pharmaceutical companies. The clinical performance of Ras farnesylation inhibitors has remained disappointing [28]. Several kinase inhibitors targeting this pathway are also undergoing clinical trials. In this context, COB223 appears as a promising leader compound targeting the Ras pathway by a novel mechanism.

## MATERIALS & METHODS

### Cell culture, small molecules and reagents

HMEC-1, HMEC-GFP, NIH-3T3, and 3T3-GFP were maintained in DMEM 1g/L glucose (Invitrogen) supplemented with 10% foetal calf serum (FCS). Human dermal microvascular endothelial cells (HMVEC-d) and human umbilical vein endothelial cells (HUVEC) (Lonza) were maintained in endothelial growth medium (EGM-2-MV, Cambrex) supplemented with 5% FCS and additives recommended by the manufacturer. LLC/2 tumor cells were grown in DMEM 4.5g/L glucose (Invitrogen) supplemented with 10% foetal calf serum (FCS). CJ7 embryonic stem cells were cultured on 1% gelatin-coated dishes in Iscove's medium containing Glutamax (Iscove's modified Dulbecco's medium; Invitrogen) and supplemented with 15% FCS, 1% non-essential amino-acids, 1% ATAM, 150 μM monothioglycerol and 1000 U/mL of leukemia inhibitory factor (Chemicon). MCF-10A cells stably overexpressing oncogenic Ras^G12V^ were kindly provided to us by Dr O. Filhol (BCI lab, Grenoble, France). CCL-39 fibroblasts expressing a constitutively active Raf in an estrogen-responsive manner [29] were a generous gift from Dr G. Pagès (IRCAN, Nice, France). Unless otherwise indicated, antibodies were purchased from Cell Signaling, except for anti-phospho-MAPK (Promega), anti-MAPK (Sigma) and anti-c-Raf (BD Biosciences).

### Chemical synthesis of COB223 and analogs

W7, monodansylcadaverine, 1,2-ethylenediamine, 1,3-diaminopropane, 1,4-diaminobutane were purchased from Sigma-Aldrich (St Louis).

#### General information

Melting points were determined using a Reicher Thermovar apparatus and are uncorrected. NMR spectra were recorded on Bruker AM 300 and 400 spectrometers using solvent as the internal reference (DMSO-d6 at 2.49 ppm, CDCl_3_ at 7.24 ppm); the chemical shifts are reported in ppm, in δ units. Mass spectra were recorded on a Polarisq Thermo Finnigan spectrometer. Elemental analyses were performed at “Service de microanalyse, Université Joseph Fourier”. Reversed-phase HPLC was performed with a μ-bondapak-C18 analytical column (Waters Associates). A Waters chromatographic system was used, with two M-510 pumps and a photodiode array detector Waters 996 using Millenium 32 software. A linear gradient from 0 to 100% methanol in H_2_O pH 2.5 (phosphoric acid), 2 mL/min flow rate, was used. All starting reagents were commercially available

#### General procedure A

Stoichiometric amount (0.65 mmol) of the acyl chloride and the alkylamine (RNH_2_) were mixed together in dichloromethane (3 ml) and the resulting solution was stirred for 15 h at room temperature. Aqueous sodium carbonate solution (1M, 3 ml) was added and the resulting mixture was vigorously stirred for 1 h and then let on standing to allow clean separation of the two layers.

The organic layer was decanted, washed several times with water, and finally with brine. The organic layer was dried on MgSO_4_, filtered and evaporated to dryness under reduced pressure. Most of the newly synthesised molecules were isolated as oily residues.

The purity of the new compounds was checked by analytical hplc, and the structures confirmed by NMR analysis.

This procedure was performed with carboxylic acid chloride (benzoyl chloride), sulfonic acid chlorides (tosyl-, naphthalene sulfonyl and dansyl chlorides) or chloroformates. (para-nitrobenzyloxycarbonyl chloride).

#### General procedure B

A mixture of the amine (1.5 eq.) and naphthalic anhydride (1 eq.) in EtOH was refluxed for 24 h. The solvent was then evaporated and the residue was dissolved in the minimum amount of AcOEt. A large volume of Et_2_O was added and the resulting solid was filtered off. The organic phase was evaporated to dryness to give the desired product.

#### General procedure C (Boc deprotection)

The Boc protected amines were dissolved in EtOH (2 ml) and 37% HCl (2 ml) was slowly added to the solution cooled in an ice-bath. The solution was then kept for 1 h at rt and the solid that had formed was filtered off. To obtain the free base, the solid was dissolved in the minimum amount of water and the solution was basified by slowly adding a saturated solution of NaHCO_3_ until reaching pH 11. The aqueous solution was then extracted three times with CH_2_Cl_2_. The organic layer was washed with water and brine, dried over MgSO_4_ and evaporated.

#### *tert*-Butyl 3-(4-(3-aminopropyl)piperazin-1-yl) propylcarbamate 1

**Figure:**
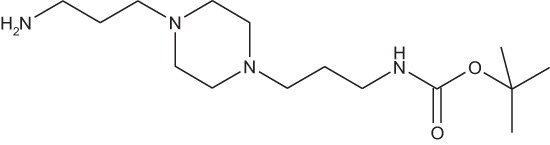


The 1,4-bis(3-aminopropyl)piperazine in large excess (4.5 ml, 21 mmol) was diluted with CHCl_3_ (25 ml). A solution of Boc_2_O (0.94 g, 4.3 mmol) diluted in CHCl_3_ (25 ml) was slowly added to the polyamine solution cooled in an ice bath and vigorously stirred. The solution was stirred at rt overnight. The solution was filtered and the solvent evaporated to dryness under reduced pressure. Saturated NaCl aqueous solution (50 ml) was added to the resulting oil to precipitate the bis-Boc protected polyamine, that was filtered off and washed with NaCl solution. The aqueous phase was then extracted three time with Et_2_O. The organic phase was then dried over MgSO_4_ and evaporated to give 1 as an oily residue (700 mg, 2.33 mmol, 54% yield).

The ^1^H-NMR spectrum was found identical to that of the commercial reagent.

^1^H-NMR (CDCl_3_) δ : 5.47 (s, 1H, NH), 3.13–3.19 (m, 2H, CH_2_NBoc), 2.72–2.76 (m, 2H, CH_2_NH_2_), 2.35–2.45 (m, 4H), 1.58–1.67 (m, 4H), 1.41 (s, 9H, CH_3_).

#### *tert*-Butyl 3-(4-(3-(5-(dimethylamino)naphthalene-1-sulfonamido)propyl)piperazin-1-yl) propylcarbamate; COB223

**Figure:**
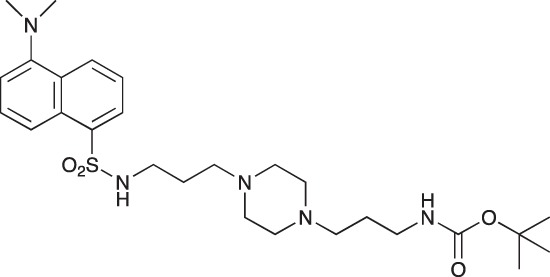


The 1,4-bis(3-aminopropyl)piperazine in large excess (3 ml, 14 mmol) was diluted with CH_2_Cl_2_ (120 ml). A solution of dansyl chloride (0,3 g, 1.15 mmol) diluted in CH_2_Cl_2_ (30 ml ) was slowly added to the polyamine solution cooled in an ice bath and vigorously stirred. The solution was stirred at rt overnight. The solution was then extracted with aqueous 1M citric acid solution. The aqueous phase was separated and basified by adding concentrated NaOH solution. Extraction of the basic solution with CH_2_Cl_2_ afforded the mono-dansyl polyamine after evaporation of the organic solvent (342 mg, 0.78 mmol, 71% yield).

The oily residue was diluted in CH_2_Cl_2_ (50 ml) and Boc_2_O (0.21 g g, 1 mmol) was added. The solution was stirred overnight, then washed once with 1N NaHCO_3_ and twice with water. The organic phase was then dried over MgSO_4_ and evaporated to give 267 mg of gummy residue that solidified upon standing (0.5 mmol, 64% yield).

^1^H-NMR (CDCl_3_) δ 8.51 (m, 1H), 8.31 (m, 1H), 8.23 (m, 1H), 7.47–7.53 (m, 2H), 7.16 (m, 1H), 5.35 (broad s, NH), 3.16–3.22 (m, 2H), 2.94 (m, 2H), 2.3–2.5 (broad m, 8H), 1.57–1.66 (m, 4H), 1.43 (s, 9H); ^13^C-NMR (CDCl_3_) δ 156.2, 152.1, 134.8, 130.2, 130.0, 129.8, 128.0, 123.3, 119.0, 115.1, 58.1, 56.7, 53.1, 45.5, 44.3, 40.0, 28.5, 26.4, 23.8

#### *tert*-Butyl 3-(4-(3-(naphthalene-1-sulfonamido)propyl)piperazin-1-yl) propylcarbamate; COB235

**Figure:**
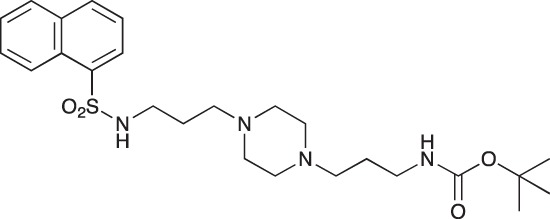


COB235 was prepared following method A from 1 (200 mg, 0.65 mmol) and naphthyl sulfonic chloride (147 mg, 0.65 mmol). COB235 was isolated as an oil (239 mg, 75% yield).

^1^H-NMR (CDCl_3_) δ 8.59–8.62 (m, 1H), 8.15–8.17 (m, 1H), 7.95–7.97 (m, 1H), 7.85–7.87 (m, 1H), 7.43–7.54 (m, 3H), 5.37 (broad s, 1H), 5.21 (broad s), 3.11–3.13, (m, 2H), 2.87 (m, 2H), 2.21–2.39 (broad m, 8H), 1.50–1.62 (m, 4H), 1.48 (s, 9H); ^13^C-NMR (CDCl_3_) δ 156.0, 134.6, 134.2, 133.9, 129.7, 129.1, 128.2, 127.9, 126.7, 124.5, 124.2, 57.8, 56.5, 53.0, 44.0, 39.7, 28.5, 26.4, 23.8

#### *tert*-Butyl 3-(4-(3-phenylamidopropyl)piperazin-1-yl)propylcarbamate; COB237

**Figure:**
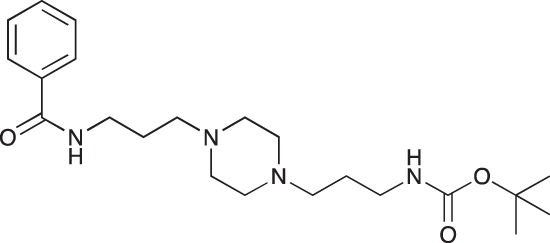


COB237 was prepared following method A from 1 (200 mg, 0.65 mmol) and benzoyl chloride (76 μl, 0.65 mmol). COB237 was isolated as an oil (171 mg, 65% yield)

^1^H-NMR (CDCl_3_) δ 8.21 (broad s, 1H), 7.77–7.81 (m, 2H), 7.35–7.47 (m, 3H), 5.36 (broad s, 1H), 3.51 (m, 2H), 3.13 (m, 2H), 2.34–2.55 (m, 8H), 1.75 (m, 2H), 1.61 (m, 2H), 1.41 (s, 9H); ^13^C-NMR (CDCl_3_) δ 167.3, 156.0, 134.9,131.2, 128.3, 127.1, 58.3, 56.8, 53.4, 53.1, 40.8, 39.7, 28.4, 26.4, 24.3

#### *tert*-Butyl 3-(4-(3-(4-methylphenylsulfonamido)propyl)piperazin-1-yl) propylcarbamate; COB236

**Figure:**
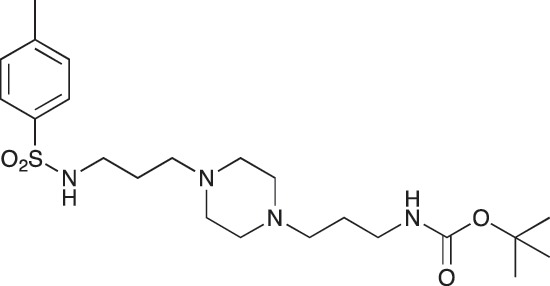


COB236 was prepared following method A from 1 (200 mg, 0.65 mmol) and para-toluene sulfonyl chloride (124 mg, 0.65 mmol). COB236 was isolated as an oil (197 mg, 66% yield).

^1^H-NMR (CDCl_3_) δ 7.71 (d, 2H), 7.28 (d, 2H), 5.4 (broad s, 1H), 3.14–3.20 (m, 2H), 3.03 (t, 2H), 2.36–2.44 (m, 13H), 1.56–1.68 (m, 4H), 1.42 (s, 9H); ^13^C-NMR (CDCl_3_) δ 156.1, 143.0, 137.2, 129.6, 127.0, 57.9, 56.5, 53.0, 44.1, 39.8, 28.4, 26.3, 24.0, 21.5

#### 4-Nitrobenzyl N-[3-(4-{3-[(tert-butoxycarbonyl)amino]propyl}piperazin-1-yl)propyl] carbamate; COB238

**Figure:**
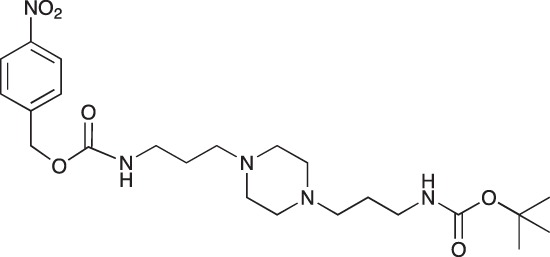


COB238 was prepared following method A from 1 (200 mg, 0.65 mmol) and 4-nitrobenzyl chloroformate (140 mg, 0.65 mmol). COB238 was isolated as an oil (196 mg, 63% yield)

^1^H-NMR (CDCl_3_) : δ 8.19 (d, 2H), 7.48 (d, 2H), 6.16 (broad s, NH), 5.36 (broad s, NH), 5.17 (s, 2H), 3.28 (quad, 2H), 3.13–3.19 (m, 2H), 2.37–2.46 (m, 8H), 1.59–1.72 (m, 4H), 1.42 (s, 9H); ^13^C-NMR (CDCl_3_) δ 156.2, 147.0, 144.5, 128.1, 127.1, 123.8, 64.9, 63.9, 57.1, 56.8? 53.2, 41.0, 39.9, 28.5, 26.4, 25.6

#### 5-(Dimethylamino)-N-[3-(dimethylamino)propyl]naphthalene-1-sulfonamide; COB221

**Figure:**
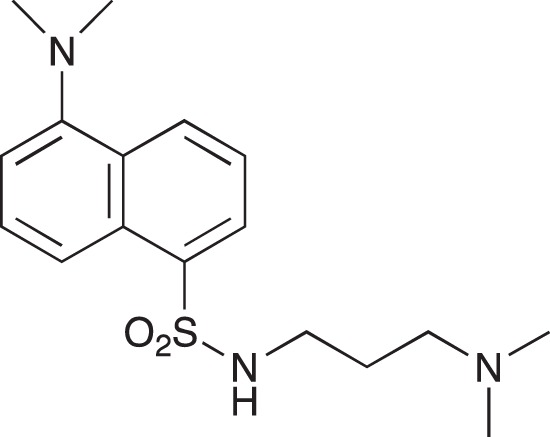


COB221 was prepared following method A from N,N-diaminopropylamine (314 μl, 2.5 mmol) and dansyl chloride (270 mg, 1 mmol). COB221 was isolated as an oil. The ^1^NMR spectrum was found identical to the literature data [30].

^1^H-NMR (CDCl_3_) : δ 8.49–8.52 (m, 1H), 8.28–8.31 (m, 1H), 8.21–8.23 (m, 1H), 7.49–7.56 (m, 2H), 7.16 (d, 1H), 2.95 (t, 2H), 2.87 (s, 6H), 2.21 (t, 2H), 2.16 (t, 6H), 1.55 (quint, 2H)

#### 5-(Dimethylamino)-N-[3-hydroxypropyl]naphthalene-1-sulfonamide; COB220

**Figure:**
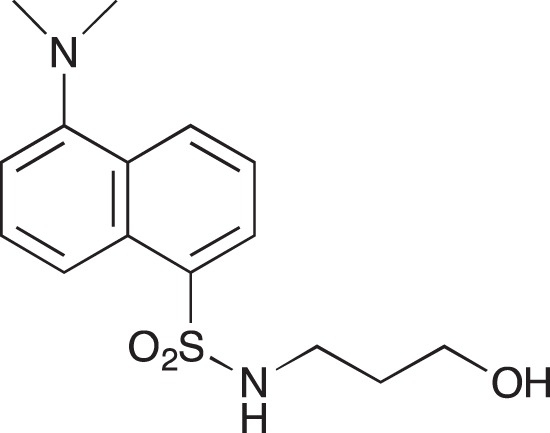


COB220 was prepared following method A from 3-aminopropan-1-ol (0.19 ml, 2.5 mmol) and dansyl chloride (270 μγ, 1 mmol). The ^1^NMR spectrum was found identical to that of the commercial reagent.

^1^H-NMR (CDCl_3_) : δ 8.56 (d, 1H), 8.24–8.31 (m, 2H), 7.50–7.60 (m, 2H), 7.20 (d, 1H), 5.20 (m, 1H), 3.66 (broadm, 2H), 3.06 (quad, 2H), 2.80 (s, 6H), 1.64 (quint, 2H)

#### 2-(2-{4-[5-(Dimethylamino)naphthalene-1-sulfonyl]piperazin-1-yl}ethoxy)ethan-1-ol; COB224

**Figure:**
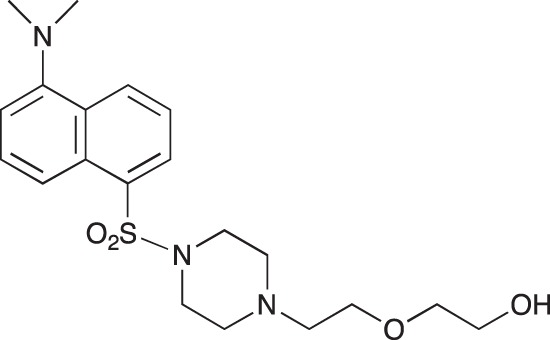


COB224 was prepared following method A from 2-[2-(1-piperazinyl)ethoxy]ethanol (0.5 ml, 2.9 mmol) and dansyl chloride (270 mg, 1 mmol). COB237 was isolated as an oil (294 mg, 0.72 mmol, 72%)

^1^H-NMR (CDCl_3_) : δ 8.55 (m, 1H), 8.41 (m, 1H), 8.17 (m, 1H), 7.48–7.54 (m, 2H), 7.16, (m, 1H), 3.49–3.62 (m, 6H), 3.22–3.25 (m, 4H), 2.87 (s, 6H), 2.53–2.57 (m, 6H); ^13^C-NMR (CDCl_3_) δ 151.8, 132.8, 130.7, 130.6, 130.5, 130.2, 128.1, 123.2, 119.8, 115.3, 72.3, 67.8, 61.9, 57.5, 52.8, 45.5, 45.4

#### 2-{2-[5-(Dimethylamino)naphthalene-1-sulfonamido]ethoxy}ethan-1-ol; COB225

**Figure:**
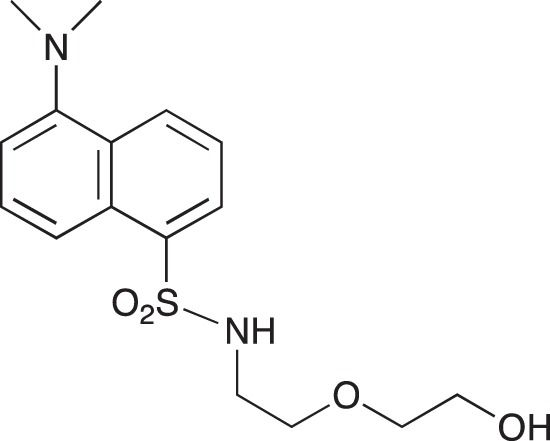


COB225 was prepared following method A from 2-[2-aminoethoxy]ethanol (0.5 ml, 4.9 mmol) and dansyl chloride (270 mg, 1 mmol). COB237 was isolated as an oil (239 mg, 0.70 mmol, 70%)

^1^H-NMR (CDCl_3_) : δ 8.57 (d, 1H), 8.25–8.32 (m, 2H), 7.5–7.6 (m, 2H), 7.21 (d, 1H), 5.25 (m, 1H, NH), 3.57 (t, 2H), 3.39 (t, 2H), 3.31 (t, 2H), 3.13, (m, 2H), 2.91 (s, 6H); ^13^C-NMR (CDCl_3_) δ 1519, 135.1, 130.5, 129.9, 129.7, 129.5, 128.4, 123.4, 119.0, 114.4, 72.2, 69.2, 61.6, 45.5, 43.2

#### 5-Dimethylamino-N-(2-[2-{2-(5-(dimethylamino)naphthalene-1-sulfonamido)ethoxy}ethoxy] ethyl)naphthalene-1-sulfonamide; COB295

**Figure:**
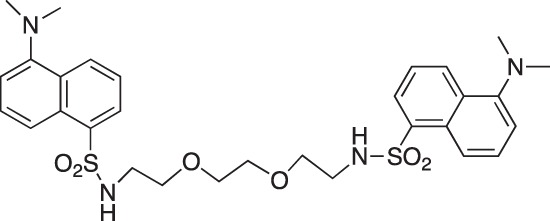


COB295 was prepared by mixing dansyl chloride (278 mg, 1.03 mmol) and 2-[2-(2aminoethoxy)ethoxy]ethan-1-amine (60 μl, 0.4 mmol) in dioxane (5 ml). The solution was stirred overnight at rt. The solvent was evaporated and the residue diluted with CH_2_Cl_2_ and pH 10 aqueous solution. The organic phase was separated, washed with water and brine, dried over MgSO_4_ and the solvent was evaporated. COB295 was obtained as an oil, (59 mg, 0.09 mmol, 24% yield.

^1^H-NMR (CDCl_3_) : δ 8.46 (m, 2H), 8.22 (m, 2H), 8.15 (m, 2H), 7.40–7.47 (m, 4H), 7.10 (m, 2H), 5.44 (br m, 2NH), 3.26–3.29 (m, 4H), 3.15 (m, 4H), 2.9–3.03 (m, 4H), 2.81 (s, 12H).

#### *tert*-Butyl N-(2-[2-{2-(5-(dimethylamino)naphthalene-1-sulfonamido)ethoxy}ethoxy]ethyl) carbamate; COB296

**Figure:**
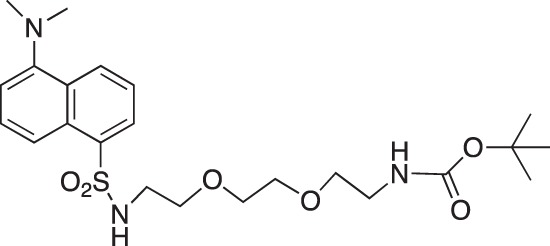


COB296 was prepared following the procedure reported in the literature from dansyl chloride (200 mg, 0.74 mmol) and N-8-tert-butoxycarbonylamine-3, 6-dioxaoctylamine (227 mg, 0.91 mmol). COB296 was obtained as a gum (191 mg, 0.39 mmol, 53% yield). The NMR data was identical to the literature data [31].

^1^H-NMR (CDCl_3_) : δ 8.59–8.51 (m, 1H), 8.36–8.38 (m, 1H), 8.29–8.31 (m, 1H), 7.55–7.64 (m, 2H), 5.48 (br s, NH), 5.04 (br s, 1H), 3.15–3.54 (m, 10H), 3.15–3.19 (m, 2H), 2.29 (s, 6H), 1.48, (s, 9H)

#### N-{3-[4-(3-*tert*-Butoxycarbonylaminopropyl)piperazin-1-yl]propyl}-1,8-naphthalimide; COB227

**Figure:**
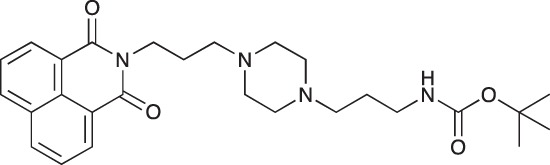


COB227 was obtained using General Procedure B from naphthalic anhydride (1 g, 5 mmol) and 1 (1.87 g, 6.25 mmol). COB227 was isolated as a solid (2.05 g, 84% yield).

Mp 196–198°C; ^1^H-NMR (300Hz, CDCl_3_) : δ 8.60–8.63 (m, 2H), 8.21–8.24 (m, 2H), 7.75–7.80 (m, 2H), 5.44 (s, 1H, NH), 4.42–4.30 (m, 2H), 3.15–3.10 (m, 2H), 2.50–2.57 (m, 8H), 2.38–2.41 (m, 4H), 1.92–2.06 (m, 2H), 1.60–1.69 (m, 2H), 1.44 (s, 9H); ^13^C-N (100Hz, CDCl_3_): δ 164.4, 156.2, 134.0, 131.7, 131.2, 128.3, 127.0, 122.9, 56.9, 56.2, 53.1, 53.1, 40.1, 39.0, 28.5, 26.3, 25.1; MS : 481.3 [*M*+H]

#### N-(3-(*tert*-Butoxycarbonylamino)propyl)-1,8-naphthalimide; COB283

**Figure:**
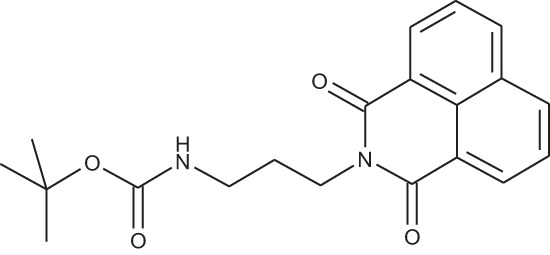


COB283 was obtained using General Procedure B from naphthalic anhydride (99 mg, 0.5 mmol) and 3-(N-tert-butoxycarbonylamino)propyl amine (108 mg, 0.6 mmol). COB283 was isolated as a solid (163 mg, 92% yield)

Mp 119–120°C; ^1^H-NMR (DMSO-d6) : δ 8.49 (m, 2H), 8.45 (m, 2H), 7.86 (m, 2H), 6.8 (br s, 1H, NH), 4.05 (t, 2H), 2.92 (m, 2H), 1.76 (quint, 2H), 1.35 (s, 9H); ^13^C-NMR (CDCl_3_) : δ 164.6, 156.1, 134.1, 131.6, 131.4, 128.2, 127.1, 122.5, 38.1, 37.7, 28.6, 28.5; MS : 377.2 [*M*+Na]

#### N-(3-Aminopropyl)-1,8-naphthalimide hydrochloride; COB226

**Figure:**
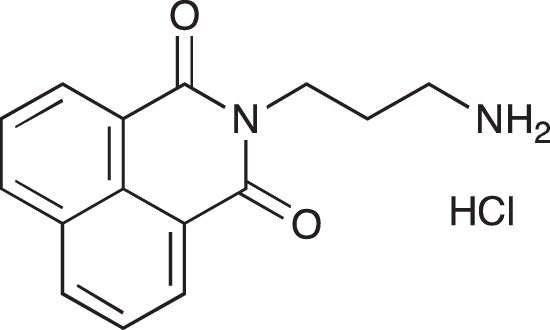


COB226 was prepared from the corresponding tert-butoxycarbonyl (Boc) protected amine by hydrolysis following General Method C in agreement with a reported procedure [32]. Yield: 85%, of hydroscopic residue

Mp 298–299°C; ^1^H-NMR (DMSO-d6): δ 8.49–8.52 (m, 2H), 7.96 (broad s), 8.46–8.49 (m, 2H), 7.86–7.91 (m, 2H), 4.10–4.15 (m, 2H), 2.79–3.04 (m, 2H), 1.94–2.03 (quint, 2H) ; ^13^C-NMR (CDCl_3,_ free base): δ 163.7, 134.3, 131.3, 130.7, 127.4, 127.2, 122.1, 37.0, 36.9, 25.9; MS : 255.1 [*M*+H], 277.1 [*M*+Na]

#### N-{3-[4-(3-Aminopropyl)piperazin-1-yl]propyl}-1,8-naphthalimide hydrochloride; COB228

**Figure:**
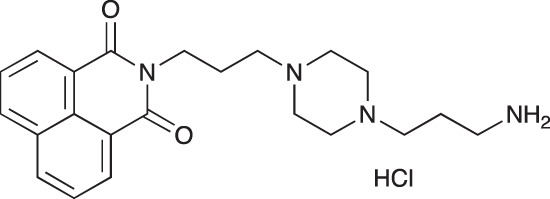


COB228 was obtained either from COB227 following General Method C, or from naphthalic anhydride and a five fold excess of 1,4-bis(3-aminopropyl)piperazine following a reported procedure [33].

Hygroscopic solid.

^1^H-NMR (DMSO-d6): δ 8.46–8.52 (m, 4H), 8.12 (broad s, 2H), 7.86–7.91 (m, 2H), 4.12–4.16 (t, 2H), 3.2–3.6 (broad m, 14H), 2.14–2.16 (m, 2H), 1.98–2.00 (m, 2H)

#### N-{3-[4-(3-*tert*-Butoxycarbonylaminopropyl)piperazin-1-yl]propyl}-3-nitro-1,8-naphthalimide; COB280

**Figure:**
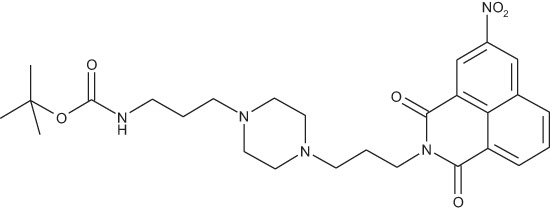


COB280 was obtained using General Procedure B from 3-nitro-naphthalic anhydride (243 mg, 1 mmol) and 1 (373 mg, 1.2 mmol). COB280 was isolated as an oil (480 mg, 93% yield)

^1^H-NMR (CDCl_3_) : δ 9.3 (d, 1H, *J* 2.1Hz, Ar), 9.1 (d, 1H, *J* 2.1Hz, Ar), 8.77 (dd, 1H, *J* 0.9 and 4.8Hz), 8.43 (m, 1H), 7.94 (m, 1H), 5.42 (br s, 1H, NH), 4.27 (t, 2H), 3.14 (m, 2H), 2.35–2.53 (m, 8H), 2.28 (m, 4H), 1.96 (m, 2H), 1.58 (m, 2H), 1.42 (s, 9H, Boc); ^13^C-NMR (CDCl_3_) : δ 163.2, 162.6, 156.1, 146.5, 135.6, 134.4, 131.1, 130.3, 129.2, 128.9, 124.9, 124.2, 123.4, 56.9, 56.1, 53.2, 46.2, 40.0, 39.5, 28.5, 26.4, 24.9; MS : 526.3 [*M*+H]

#### N-[3-((3-*tert*-Butoxycarbonylaminopropyl)amino)propyl]-3- nitro-1,8-naphthalimide; COB281

**Figure:**
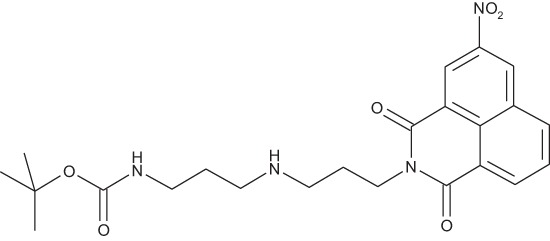


COB281 was obtained using General Procedure B from 3-nitro-naphthalic anhydride (243 mg, 1 mmol) and 3-[(3-(N-tert-butoxycarbonylamino)propyl)amino]propylamine (290 mg, 1.2 mmol). COB281 was isolated as an oil (235 mg, 51% Yield)

^1^H-NMR (DMSO-d6) : δ 9.46 (s, 1H), 8.95 (d, 1H, *J* 2.28Hz), 8.77 (m, 1H), 8.67 (m, 1H), 8.05 (m, 1H), 6.74 (br s, 1H, NH), 4.21 (t, 2H), 2.93 (m, 2H), 2.44 (m, 2H), 1.77 (m, 2H), 1.46 (m, 2H), 1.35 (s, 9H, Boc); ^13^C-NMR (CDCl_3_) : δ 163.3, 162.7, 156.3, 146.5, 135.7, 134.6, 131.1, 130.3, 129.3, 124.7, 124.4, 123.2, 53.8, 47.4, 47.0, 39.0, 38.8, 29.8, 28.5, 28.1; MS : 457.3 [*M*+H]

#### N-(3-(*tert*-Butoxycarbonylamino)propyl)-3-nitro-1,8-naphthalimide; COB282

**Figure:**
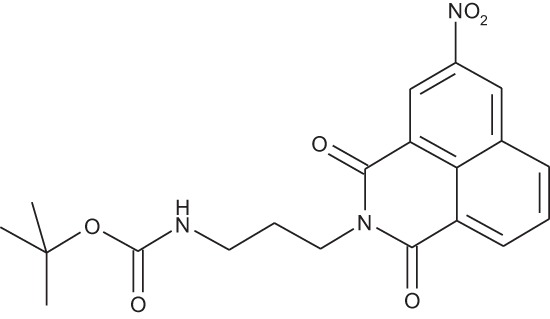


COB282 was obtained using General Procedure B from 3-nitro-naphthalic anhydride (121 mg, 0.5 mmol) and 3-(N-tert-butoxycarbonylamino)propylamine (108 mg, 0.62 mmol). COB282 was isolated as a solid (137 mg, 68% Yield)

Mp 140–141°C; ^1^H-NMR (DMSO-d6) : δ 9.46 (d, 1H, *J* 2.4Hz), 8.94 (d,1H, *J* 1.8Hz), 8.77 (m, 1H), 8.67 (m, 1H), 7.05 (m, 1H), 6.82 (br s, 1H, NH), 4.06 (t, 2H), 3.03 (quat, 2H), 1.78 (m, 2H), 1.36 (s, 9H, Boc); ^13^C-NMR (CDCl_3_) : δ 163.3, 162.8, 155.9, 146.4, 135.7, 134.6, 131.0, 130.2, 129.1, 124.4, 124.2, 123.4, 38.1, 37.6, 28.4, 28.4; MS : 422.2 [*M*+Na]

#### N-(3-Aminopropyl)-3-nitro-1,8-naphtalimide hydrochloride COB277

**Figure:**
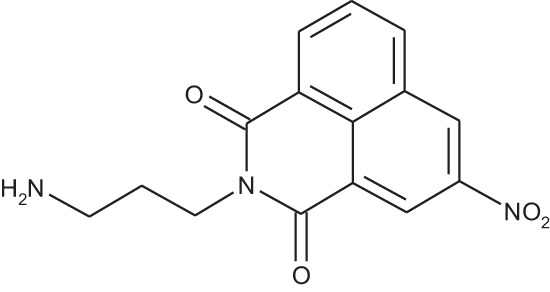


COB277 was prepared from the corresponding tert-butoxycarbonyl (Boc) substituted COB282 following General Method C. Yield: 88%, of hydroscopic residue

Mp 263–265°C

^1^H-NMR (DMSO-d6) : δ 9.48 (d, 1H, *J* 2.3Hz), 8.94 (d, 1H, *J* 2.3Hz), 8.77 (dd, 1H, *J* 0.8 and 8.4), 8.68 (dd, 1H, *J* 1.1 and 7.3), 8.06 (m, 1H), 4.13 (t, 2H), 2.87 (m, 2H), 1.98 (m, 2H); ^13^C-NMR (CDCl_3_) : δ 163.0, 162.5, 145.8, 136.3, 133.8, 130.8, 129.6, 129.6, 129.2, 124.1, 122.7, 122.6, 37.4, 36.9, 25.8; MS : 300.1 [*M*+Na]

#### N-{3-[4-(3-*tert*-Butoxycarbonylaminopropyl)piperazin-1-yl]propyl}-4-dimethylamino-1,8-naphthalimide; COB274

**Figure:**
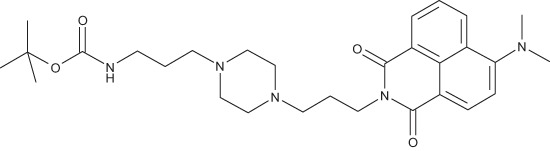


COB274 was obtained using General Procedure B from 4-dimethylamino-naphthalic anhydride (80 mg, 0.33 mmol) and 1 (132 mg, 0.4 mmol). COB274 was isolated as an oil (170 mg, 98% yield)

^1^H-NMR (DMSO-d6) : δ 8.51 (dd, 1H, *J* 1.05 and 8.52Hz, Ar), 8.43 (dd, 1H, *J* 0.99 and 7.29Hz), 8.34 (d, 1H), 7.75 (quat, 1H, *J* 1.05 and 7.87 Hz), 7.21 (m, 1H), 6.76 (s, 1H, NH), 4.06 (m, 2H), 3.31–3.46 (m, 8H), 3.08 (s, 6H, 2 × CH_3_), 2.94 (m, 2H), 2.55 (m, 4H), 1.75 (quint, 2H), 1.51 (quint, 2H), 1.34 (s, 9H, Boc); ^13^C-NMR (CDCl_3_) : δ 164.6, 164.1, 156.9, 156.1, 132.5, 131.1, 130.6, 125.3, 124.9, 123.2, 115.1, 113.3, 56.7, 56.0, 53.0, 52.8, 44.7, 39.8, 38.6, 28.4, 26.0, 24.0; MS : 524.4 [*M*+H]

#### N-(3-(*tert*-Butoxycarbonylamino)propyl)-4-dimethylamino-1,8-naphthalimide; COB276

**Figure:**
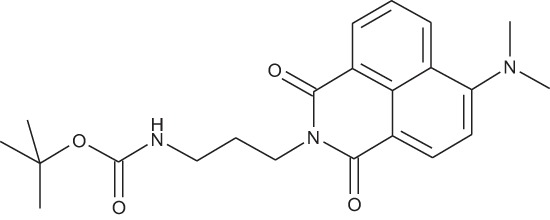


COB276 was obtained using General Procedure B from 4-dimethylamino-naphthalic anhydride (80 mg, 0.33 mmol) and 3-(N-tert-butoxycarbonylamino)propylamine (82 mg, 0.4 mmol). COB276 was isolated as a solid (130 mg, 99% Yield).

Mp 129–132°C; ^1^H-NMR (DMSO-d6) : δ 8.57 (dd,1H, *J* 0.99 and 7.26 Hz), 8.48 (m, 1H), 8.44 (m, 1H), 7.67 (m, 1H), 7.13 (m, 1H), 4.26 (t, 2H), 3.16 (m, 2H), 3.12 (s, 6H, 2 × CH_3_), 1.90 (quint, 2H), 1.45 (s, 9H, Boc); ^13^C-NMR (CDCl_3_) : δ 165.0, 164.5, 157.2, 156.1, 132.9, 131.5, 131.3, 130.4, 125.4, 125.0, 123.0, 114.8, 113.4, 53.5, 44.9, 37.4, 28.6, 28.6

MS : 398.2 [*M*+H], 420.2 [*M*+Na]

#### N-[3-((3-*tert*-Butoxycarbonylaminopropyl)amino)propyl]-4-dimethylamino-1,8-naphthalimide; COB275

**Figure:**
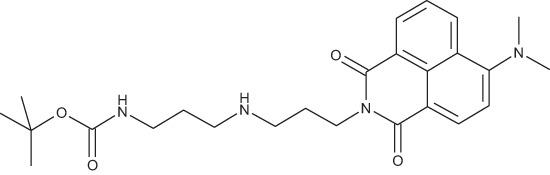


COB275 was obtained using General Procedure B from 4-dimethylamino-naphthalic anhydride (169 mg, 0.7 mmol) and 3-[(3-(N-tert-butoxycarbonylamino)propyl)amino] propylamine (243 mg, 1 mmol). COB275 was isolated as an oil (295 mg, 92% Yield).

^1^H-NMR (CDCl_3_) : δ 8.58 (dd, 1H, *J* 1.1 and 7.17 Hz), 8.48 (d, 1H), 8.42 (dd, 1H, *J* 1.14 and 7.98 Hz), 7.67 (m, 1H), 7.12 (m, 1H), 5.45 (s, 1H, NH), 4.29 (t, 2H), 3.29 (quat, 2H), 3.12 (s, 6H, 2 × CH_3_), 2.81 (m, 4H), 2.15 (quint, 2H), 1.85 (quint, 2H), 1.43 (s, 9H, Boc); ^13^C-NMR (CDCl_3_) : δ 165.1, 164.6, 157.4, 156.1, 133.2, 131.7, 131.5, 130.5, 125.2, 125.0, 12.8, 114.3, 113.4, 46.6, 46.4, 44.8, 38.2, 37.5, 28.6; MS : 455.3 [*M*+H]

#### Bis-[(3-nitro-1,8-naphthalimido)propyl]amine; COB278

**Figure:**
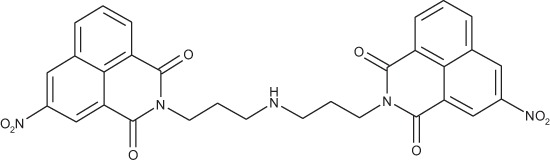


COB278 was prepared from a mixture of 3-nitro-naphthalic anhydride (304 mg, 1.2 mmol) and 1,3-bis(3-aminopropyl)amine (140 μl, 1 mmol) in dioxane (5 ml). The solution was stirred overnight at rt. The solvent was then evaporated to dryness and the residue was solubilized in a mixture of water and CH_2_Cl_2_. The organic phase was washed several time with water, brine and then dried. Evaporation of the solvent gave the desired compound as a gum (266 mg, 72% yield).

^1^H-NMR (DMSO-d6) : δ 9.4 (d, 2H, *J* 2.2Hz,), 8.94 (d, 2H, *J* 2.3Hz), 8.72 (m, 2H), 8.62 (dd, 2H, *J* 1.0 and 7.3Hz), 8.01 (m, 2H), 4.07 (t, 4H), 2.59 (t, 4H), 1.75 (quint, 4H); ^13^C-NMR (DMSO-d6) : δ 162.6, 162.0, 145.6, 136.1, 133.7, 130.6, 129.4, 129.3, 129.1, 123.8, 122.6, 122.4, 46.7, 38.5, 27.5; MS : 582.2 [*M*+H], 604.2 [*M*+Na]

#### N-[3-(5-(Dimethylamino)naphthalene-1-sulfonamido) propyl]-4-dimethylamino-1,8-naphthalimide; COB279

**Figure:**
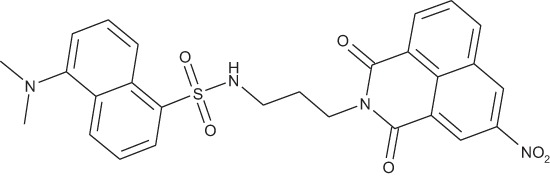


COB279 was obtained using General Procedure A from COB277 (50 mg, 0.14 mmol) and dansyl chloride (50 mg, 0.18 mmol). After purification on alumina column chromatography, COB279 was obtained as a gum (20 mg, 23%).

^1^H-NMR (DMSO-d6): δ 9.17 (d, 1H, *J* 2.2 Hz), 9.10 (d, 1H, *J* 2.1 Hz), 8.67–8.70 (dd,1H, *J* 1.1 and 7.3 Hz), 8.46–8.49 (m, 1H), 8.39–8.42 (dd,1H, *J* 0.8 and *J* 8.2 Hz), 8.38,8.40 (m,1H), 8.20–8.23 (dd,1H, *J* 1.2 and 7.2 Hz), 7.89–7.94 (m,1H), 7.61–7.67 (m,1H), 7.44–7.49 (m,1H), 7.17–7.19 (m,1H), 5.75–5.79 (br s, 1H, NH), 4.12–4.16 (t, 2H), 2.88–2.94 (m, 2H), 2.85(s, 6H, 2 × CH_3_), 1.82–1.86 (quint, 2H); ^13^C-NMR(CDCl_3_): δ 163.5, 163.0, 151.9, 146.3, 135.9, 135.2, 134.8, 131.0, 130.3, 130.1, 129.9, 129.6,129.5, 129.3, 129.2, 128.4, 124.5, 124.2, 123.2, 122.8, 119.0, 115.2, 45.4, 40.1, 37.6, 38.2; MS : 533.2 [*M*+H]

#### 1,3-bis [5-(Dimethylamino)naphthalene-1-sulfonamido] diaminopropane 05-06-L-F11

This compound was prepared from diaminopropane and an excess of dansyl chloride.

**Figure:**
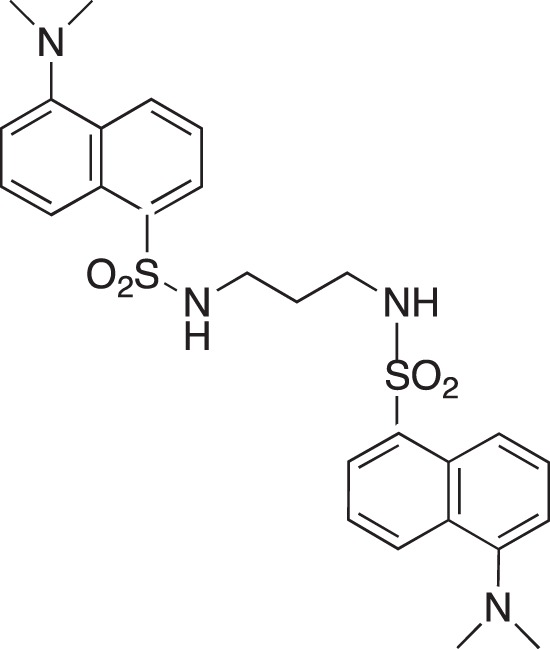


Solid, mp 90°C; ^1^H-NMR (200Hz, CDCl_3_): δ 8.40–7.00 (m, 12H), 4.80 (t, 2H), 2.80 (m, 16H), 1.35 (quint, 2H), ^13^C-NMR (50 MHz, CDCl_3_): δ 130.4, 129.4, 128.5, 123.1, 118.4, 115.2, 45.3, 39.6, 29.7; MS: m/z 540 (M^+^). Microanalysis calcd for C_27_H_32_N_4_O_4_S_2_ C 59.98, H 5.96, N 10.36; Found C 60.03, H 6.03, N 10.03

#### 1,4-bis[3-(5-(Dimethylamino)naphthalene-1-sulfonamido)propylamino]piperazine; COB222

**Figure:**
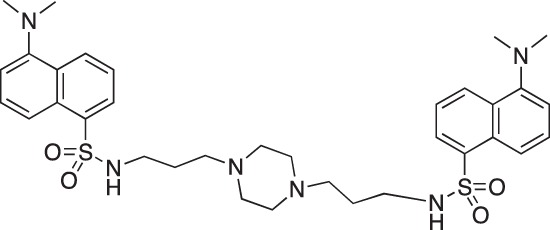


COB222 was prepared according to published procedure from a mixture of dansyl chloride (270 mg, 1 mmol) and 1,4-bis(3-aminopropyl)piperazine (82 μl, 0.4 mmol) in dioxane (5 ml). The solution was stirred overnight at rt. The solvent was then evaporated to dryness and the residue was solubilized in a mixture of 2N HCl and CH_2_CL_2_. The organic phase was removed and the aqueous phase was basified to allow precipitation of COB222. The ^1^H NMR spectrum was identical to literature data [30].

^1^H-NMR (CDCl_3_) : δ 8.48 (d, 2H), 8.30 (d, 2H), 8.11 (d, 2H), 7.90 (s, 2H), 7.60 (m, 4H), 7.28 (m, 2H), 2.85 (s, 12H), 2.80 (m, 4H), 2.05 ( broad m, 12H), 1.35 (m, 4H).

#### N-(5-(Dimethylamino)naphthalene-1-sulfonamido)-N'-(tert-butoxycarbonyl)-1,3-diamino-propane; 05-102-L-B09

**Figure:**
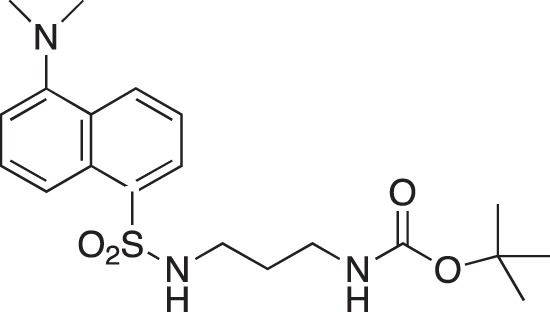


05-102-L-B09 was prepared as described previously [34].

^1^H-NMR (200Hz CDCl_3_) : δ 8.50 (d, 1H), 8.30 (d, 1H), 8.20 (d, 1H), 7.50 (m, 2H), 7.20 (d, 1H), 5.70 (br t, 1H), 4.50 (br t, 1H), 2.85 (s, 6H), 3.05 and 2.90 (2m, 4H), 1.50 (m, 2H), 1.35 (s, 9H).

#### N-(5-(Dimethylamino)naphthalene-1-sulfonamido)-1,3-diamino-propane; 05-06-L-D03

**Figure:**
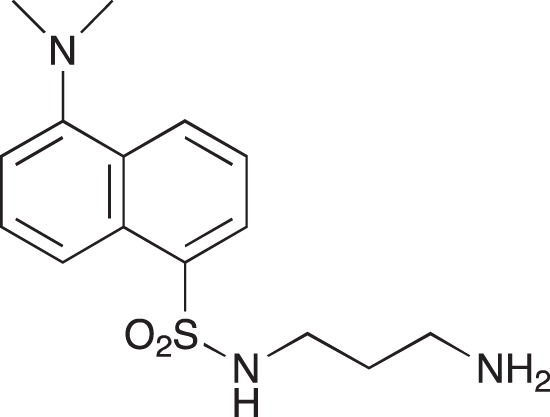


05-06-L-D03 was prepared as described previously [34].

^1^H-NMR (200Hz CDCl_3_) : δ 8.50 – 7.00 (m, 6H), 3.80 (broad m, 3H), 2.95–2.60 (m, 10H), 1.40 (q, 2H)

#### COB223-BSA conjugate

The conjugate was obtained following the route described in the following scheme.

**Figure:**
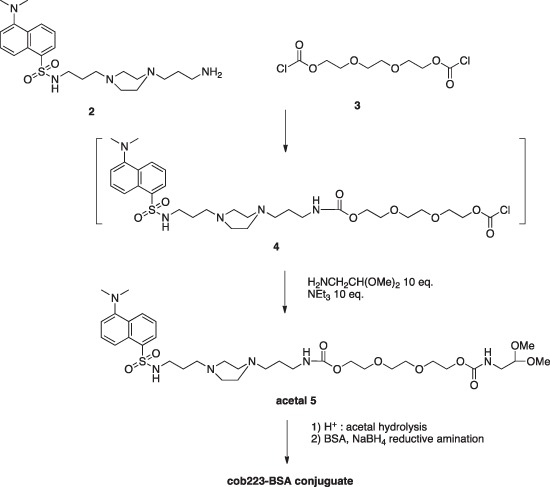


The mono-dansyl polyamine 2 (303 mg, 0.65 mmol), prepared as described above for COB223, (303 mg, 0.65 mmol) was solubilized in CH_2_Cl_2_ (20 ml) and slowly added to a solution of triethylene glycol bischloroformate 3 (1.3 ml, 6.5 mmol) in CH_2_Cl_2_ (200 ml) cooled to 0°C. The solution was stirred at rt for 30 min. Aminoacetaldehyde dimethyl acetal (1.4 ml, 13 mmol) and NEt_3_ (1.8 ml, 13 mmol) were then added and to the resulting mixture was stirred overnight at room temperature. The reaction was followed by TLC (silica gel, elution: MeOH) Diluted citric acid aqueous solution was then added to the organic solution. The water phase was separated, basified to pH 10 and extracted with CH_2_Cl_2_. The organic phase was washed with water and brine, dried over MgSO_4_ and evaporated. The fluorescent yellow oil (345 mg) thus obtained was analyzed by NMR and used in the BSA conjugation step without any further purification.

5. 1H NMR (CDCl_3_) δ 8.57–8.60 (m, 1H), 8.35–8.37 (m, 1H), 8.27–8.29 (m, 1H), 7.57–7.59 (m, 3H), 7.21–7.24 (m, 1H), 5.7 (broad s), 5.15–5.21 (broad s), 4.43 (t, 1H, CH acetal), 4.26 (broad s, 4H), 3.67–3.78 (m, 8H), 3.43 (s, 6H, 2xOCH_3_), 3.29–3.36, (m, 4H), 3.01 (m, 2H),2.94 (s, 6H, 2xNCH_3_), 2.50–2.94 (broad m, 10H), 1.50–1.62 (m, 4H)

The COB-acetal 5 (300 mg, 0.40 mmol) was diluted in aqueous 0.01M HCl (6 ml) and the pH value was adjusted to 2 by adding 1N HCl. The reaction was carried out at 20°C. The presence of the free aldehyde was checked by mixing a small aliquot of the reaction mixture to a 2,4-dinitrophenylhydrazine aqueous solution (20 mg in 5 mL). The formation of the corresponding 2,4-dinitrophenylhydrazone was evidenced as a new spot on the TLC (elution: MeOH).

An aliquot of the reaction mixture (300 μl, 0.08 mmol) was mixed with BSA (20 mg, 3.10^−7^ mol) in 2 ml of phosphate buffer (0.1 M, pH 7.0) at room temperature. Every 2h, 50 ml of a freshly prepared NaBH_3_CN aqueous solution consisting of 10 mg (0.26 mmol) dissolved in 360 ml of phosphate buffer (0.1 M, pH 7) was added to the vial. After the third addition, the solution was kept overnight at 4°C. The solution was then chromatographed through Sephadex G25 column eluted with neat water. The presence of dansyl in the fractions corresponding to the protein was checked by fluorescence (UV illumination at 365 nm) and aliquots of 10 ml were mixed with 10 ml of loading buffer (100 mM Tris-Cl, 4%SDS, 0.2% bromophenol blue, 20% glycerol, 200 mM dithiothreitol), denatured by heating at 90°C for 10 min and then analyzed by SDS polyacrylamide gel electrophoresis. The fluorescent staining of the bands corresponding to BSA confirmed the covalent bonding of COB derivative. Fractions containing exclusively the BSA-COB conjugate were pooled and filtered through 0.22 mm filter affording 4 μmol of BSA-COB conjugate (based on dansyl absorbance, ε = 4200 at 334 nm).

### Preparation of green fluorescent cell lines

HMEC-1 or NIH-3T3 cells were infected by a defective retrovirus coding for the enhanced green fluorescent protein (EGFP). A construct containing EGFP under the control of herpetic human cytomegalovirus (HCMV) promoter, and neo gene (vector pEGFP-N1, Clontech, St Germain-en-Laye, France), was subcloned in the pLNCX vector. This construct was introduced in PT67 cells by transfection with Effectene (Qiagen, Hilden, Germany). The supernatant of PT67 cells was filtrated to collect viral particles, in order to infect HMEC-1 and 3T3 cells. Neomycin-resistant clones expressing EGFP were isolated by limit dilution and amplified as independent cell lines. These cell lines stably expressing EGFP were named HMEC-GFP and 3T3-GFP.

### Quality control of the screening assay

The screening of the chemical library was performed on HMEC-GFP cell monolayers grown in 96-well plates by measuring the rate of closure of a linear wound made automatically in each well by pipette tips. To evaluate the quality of the assay, the Z' factor was calculated using the following equation according to Zhang et al. [35]:

$$Z^{'}=1-\frac{\left(3{\text{σ}}_{c+}\;+3{\text{σ }}_{c-}\right)}{\left|{\text{μ}}_{c+}\;-{\text{ μ}}_{c-}\right|}$$

where c+ are the bioactive controls (0.5% serum), c– the negative controls (10% serum), μ the mean of 48 repeats, and σ the standard deviation. Under our conditions, we calculated a Z' factor of 0.59. For cell-based assays, Z' factors greater than 0.4 are deemed acceptable. Thus, this test was compatible with high-throughput screening requirements.

### Scratch assay

Confluent cell monolayers were wounded with a plastic pipet tip, washed gently with phosphate buffer saline (PBS) and treated with complete media containing molecules, as indicated. Cells were placed at 37°C into the incubator and photographed at indicated times. Quantitation of closure of the monolayer was performed using the NIH Image J program (http://rsbweb.nih.gov/ij/). Results are expressed as percentage of wound closure at *t* = 24 h as compared to the initial wound at *t* = 0 h.

### Cell migration assay using Boyden chambers

The assay was performed using 24-multiwell plates and individual cell culture inserts bearing a porous (size of pores: 3 μm) and dyed bottom membrane, which were purchased from BD (FluoroBlok^TM^). HUVEC cells were rendered fluorescent by incubation for 24 h in the presence of 5 μg/ml of Di-I (1,1′-dioctadecyl-3,3,3′3′-tetramethylindocarbocyanine perchlorate) and subsequently seeded (5 × 10^4^ cells/insert) in the upper chambers (inserts) in serum-free culture medium supplemented with 25 μM COB223 or with vehicle (DMSO). Serum supplemented medium (5% FCS) was placed in the lower chambers (plate wells). After incubation for 4 h or 24 h at 37°C, the fluorescence of cells that had passed through the dyed membrane was measured on a plate reader at 540 nm/570 nm excitation/emission wavelengths.

### Cell proliferation

Cells were treated with molecules in complete medium. After 22 h, 1 μCi per well H^3^ thymidine was added for 2 hours. Cells are then fixed by 10% trichloroacetic acid and harvested with 0.2N NaOH, 1% SDS. Radioactivity was measured in a beta counter.

### Cell viability

Once they had reached confluency, cells were kept for 24 hours in medium supplemented with 0.5% FCS (LLC/2 and 3T3 cells) or 2.5% FCS (HUVEC and HMVEC-d cells), in the presence of COB223 at different concentrations. WST-1 assay (Roche Diagnostic, Meylan, France) was then performed according to the manufacturer's instructions.

### Sprouting of HMEC-GFP

To prepare spheroids, HMEC-GFP cells were seeded at 3000 cells/well in round-bottom 96-multiwell plates in DMEM 1 g/L glucose (Eurobio, Les Ulis, France) containing 0.25% methylcellulose. 48 h later, the spheroids were collected, transferred into flat-bottom 96-well plates and embedded in collagen gel (1.2 mg/mL type I collagen) prepared in a Iscove's modified Dulbecco's medium, supplemented with FGF-2 (10 ng/mL), 50 U/mL penicillin and 50 μg/mL streptomycin. Treatment with COB223 (10 and 20 μM) started right after spheroids were included in the collagen gels. Sprouting was allowed for 24 h at 37°C. Images were acquired by fluorescence microscopy and analysis of gel invasion was performed using the freeware Image J. Statistical analysis was performed using a Kruskal & Wallis test.

### Invasion of HMEC-GFP spheroids in collagen matrix

HMEC-GFP cells were developed as spheroids in an Iscove's modified Dulbecco's medium containing 0.2% methylcellulose in round-bottom 96-multiwell plate. Two days later, spheroids were collected and invasion was allowed in a Iscove's modified Dulbecco's medium, supplemented with 1.2 mg/mL collagen I, 50 U/mL Penicillin, 50 μg/mL streptomycin. Molecules (10 or 20 μM) or FGF-2 (10 ng/mL) were added in the preparation before collagen gelification.

### Embryonic stem cells (ESC) differentiation into endothelial lineage

CJ7 cells (1000 cells/well; 12-well plates) were allowed to differentiate in collagen gels in Iscove's medium containing Glutamax (Iscove's modified Dulbecco's medium; Invitrogen) supplemented with 1.2 mg/mL collagen I (BD Biosciences, Bedford, MA), 15% fetal calf serum (Invitrogen), 450 μM monothioglycerol (Sigma-Aldrich), 10 μg/mL insulin (Roche Diagnostics, Basel, Switzerland), 50 U/mL penicillin and 50 μg/mL streptomycin (Invitrogen), as previously described [36]. A cocktail of growth factors was also added containing 50 ng/mL human VEGF-A (AbCys) and 100 ng/mL human FGF-2 (a generous gift from Dr. A. Baird, Whittier Institute, La Jolla, CA). At day 6, molecules were added with a novel addition of 50 ng/mL human VEGF-A and 100 ng/mL human FGF-2. Differentiation was allowed during 11 days.

### Immunocytochemistry of embryoid bodies (EBs)

Collagen gels containing EBs were dehydrated and fixed in 4% paraformaldehyde during 15 min at room temperature. EBs were incubated for 1 h with a rat monoclonal anti-mouse platelet/endothelial cell adhesion molecule (PECAM)/CD31 antibody (clone MEC-13.3, a gift from Dr A. Vecchi, Milano, Italy) as previously described [37], followed by a 40 min incubation with a cyanine-3-labeled donkey anti-rat Ig antibody (Jackson Immunoresearch Laboratories, West Grove, PA). EBs were then mounted before examination under an epifluorescence microscope. For quantitation of endothelial sprouting, images were captured with a digital camera and measurement of the total length of endothelial sprouts was achieved by morphometric analysis using ImageJ software.

### Ethics statement

Mice were housed under conventional conditions in the animal care facility at the Commissariat à l'Energie Atomique et aux Energies Alternatives. All experimental procedures were conducted according to the institutional guidelines and those formulated by the European Community for the Use of Experimental Animals. The animal protocol was approved by the Institutional Animal Care and Use Committee of the Commissariat à l'Energie Atomique.

### Mouse sponge angiogenesis assay

Disc Cellspon cellulose sponges (thickness 2 mm, diameter 10 mm, Cellomeda; Turku, Finland) were implanted under the skin of Balb-c mice under general anesthesia. Mice were divided in three groups receiving either DMSO, FGF-2, or FGF-2 and COB223 (groups of respectively 6, 7 and 8 mice). The sponges were hydrated with 50 μl of PBS and 1.25% DMSO (vehicle of COB223), or FGF-2 (200 ng) or a mixture of FGF-2 (200 ng) and COB223 (100 μM). DMSO, FGF-2 and/or COB223 (50 μL) were injected again into the sponges through the skin on day 1 and 2. A third injection of COB223 alone was performed on day 4. On day 7, the vascularization of the sponges was imaged by non-invasive fluorescence imaging after i.p. injection of RAFT-(cRGD)4 (Angiostamp^TM^, Fluoptics, Grenoble, France) as described in [9]. The mice were then anesthetized and sacrificed by cervical dislocation on day 7 and the sponges were rapidly excised and photographed. Each sponge was then homogenized in 1 mL RIPA lysis buffer and the supernatants were used for quantification. The extent of vascularization of the sponge implants was assessed by measuring the amount of hemoglobin using the Drabkin's reagent (Sigma).

### Mouse LLC/2-tumor model

We chose this model because of its rapid growth in syngenic mice and its strong dependence on angiogenesis. LLC/2 cells (10^6^ in 50 μL medium) were subcutaneously injected into the right flank of nude mice. Seven days after tumor inoculation, mice were given an intraperitoneal (i.p.) injection of either solvent (1.7% DMSO) or COB223 every other day. Tumor size was measured with calipers, and the volume was calculated according to the formula (L*w^2^)/2 where L and h stand for length and width respectively. All mice were killed 21 days after tumor grafting.

### Immunohistochemistry

Tumors collected after animal sacrifice were split into two parts and fixed either in 4% paraformaldehyde (v/v) or in formalin fixative for 24 h at 4°C and embedded in paraffin and then cut into 7 μm sections. Sections were stained using specific antibodies and the avidin–biotin immunoperoxidase detection method. Endogenous peroxidase activity was quenched by pretreatment with 3% (v/v) hydrogen peroxide in methanol for 20 min. For endothelial immunohistochemistry detection, CD31 antibody (MEC13.3, BD Pharmingen) was incubated for 2 h at room temperature. The tissue sections were subsequently washed three times with PBS and incubated with biotinylated rabbit anti-rat IgG (1:250 dilution in blocking solution; Sigma-Aldrich, Saint-Quentin Fallavier, France) for 1 h at room temperature. After three PBS washes, the slides were incubated with an avidin: biotin complex (Vectastain ABC kit; Vector Laboratories, Burlingame, CA) for 45 minutes. After a final PBS wash, the immunoreactive proteins were visualized after the addition of 3,3′-diaminobenzidine (Dako, Trappes, France) for 2 min and then counterstained with hematoxylin. Control sections were treated without primary antibody.

### Western blotting

Serum-starved HUVEC cells were incubated for 15 min in the presence of either 10 ng/mL VEGF-A or 20 ng/mL FGF-2 and in the absence or the presence of indicated concentration of COB223. COB223 was incubated 15 min prior to stimulation. Cells were lysed in RIPA buffer ( Tris HCl pH 7.4 10 mM, NaCl 150 mM, SDS 0.1%, Na Deoxycholate 0.5%, EDTA 1 mM, Triton X100 1% and protease inhibitor cocktail (Sigma) for protein extraction. After fractionation of proteins on a 4–15% SDS-PAGE gel and blotting to a nitrocellulose membrane, immunoblotting detection of proteins was done using indicated antibodies according to manufacturer's instruction.

## SUPPLEMENTARY FIGURES


