# Supplementary material for: A new chemical inhibitor of angiogenesis and tumorigenesis that targets the VEGF signaling pathway upstream of Ras
Source: Oncotarget. 2015 Jan 23;6(7):5382–411. doi: 10.18632/oncotarget.2979 (PMC4467156; doi:10.18632/oncotarget.2979)
Supplement: Supplementary file 1 [file oncotarget-06-5382-s001.pdf]

SUPPLEMENTARY FIGURES

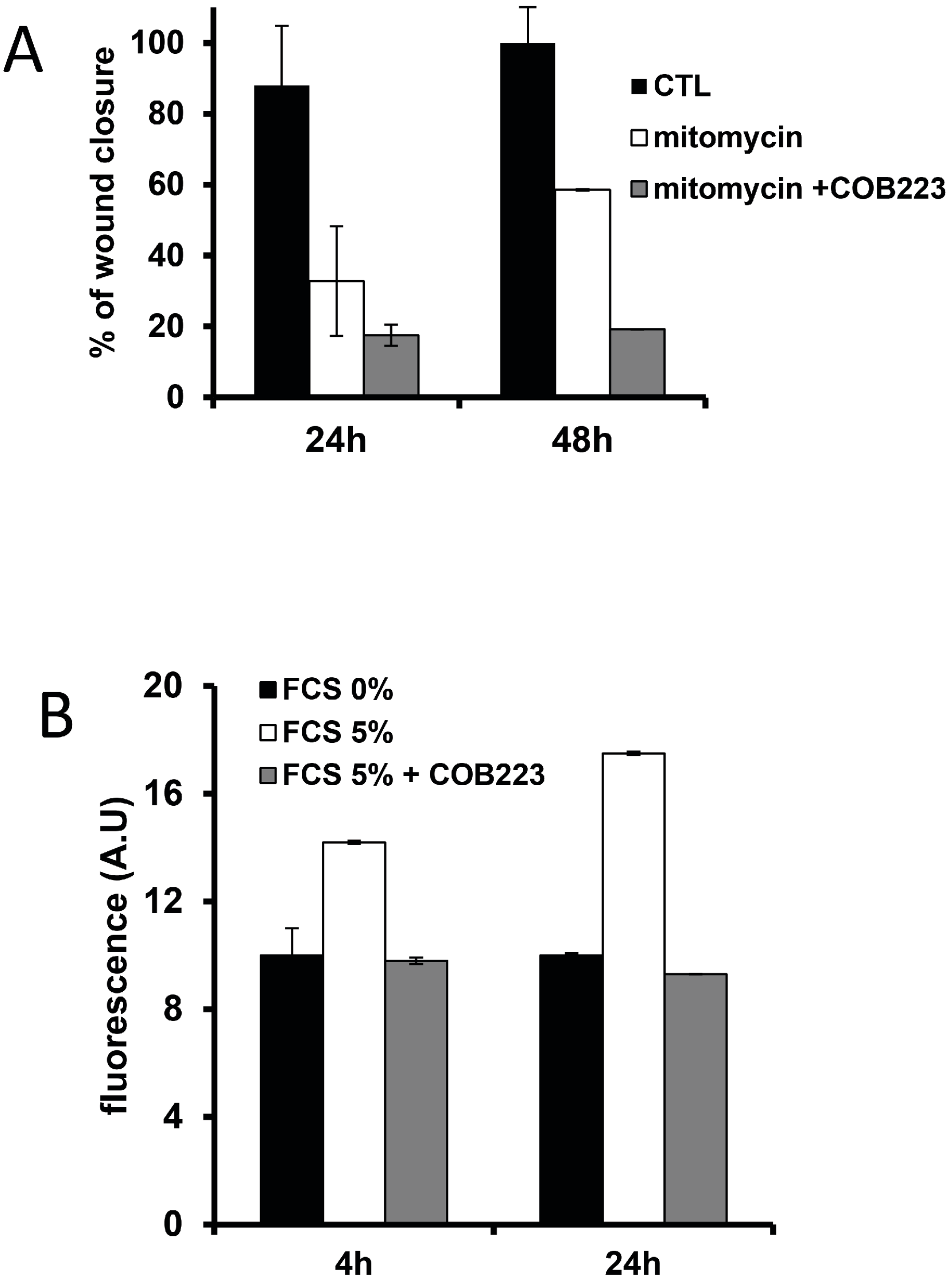

Figure S1: Effect of COB223 on endothelial cell migration. **(A)** Scratch assay: Confluent HUVEC cells were pretreated for 24 h without (CTL) or with 2 µg/mL of mitomycin C before a wound was made in the cell monolayer. After wounding, cells were incubated at 37°C in the absence or the presence of 25 µM COB223. Wound closure was measured after 24 h and 48 h of incubation as described in Material and Methods. The mean value ± SD from triplicates in two independent experiments is represented. Determination of ^{3}H-thymidine incorporation into mitomycin C-treated cells showed a complete inhibition as compared to that in untreated cells (data not shown). **(B)** Boyden chamber assay: The technique is described in Material and Methods. We measured the passage of fluorescently labeled HUVEC cells through a 3 µm-porous membrane in response to fetal calf serum used as a chemo-attractant. 25 µM COB223 or solvent (DMSO) were present in the upper chambers during the assay. The Figure represents the mean fluorescence ± SD of duplicate determinations after 24 h or 48h of incubation from two independent experiments.

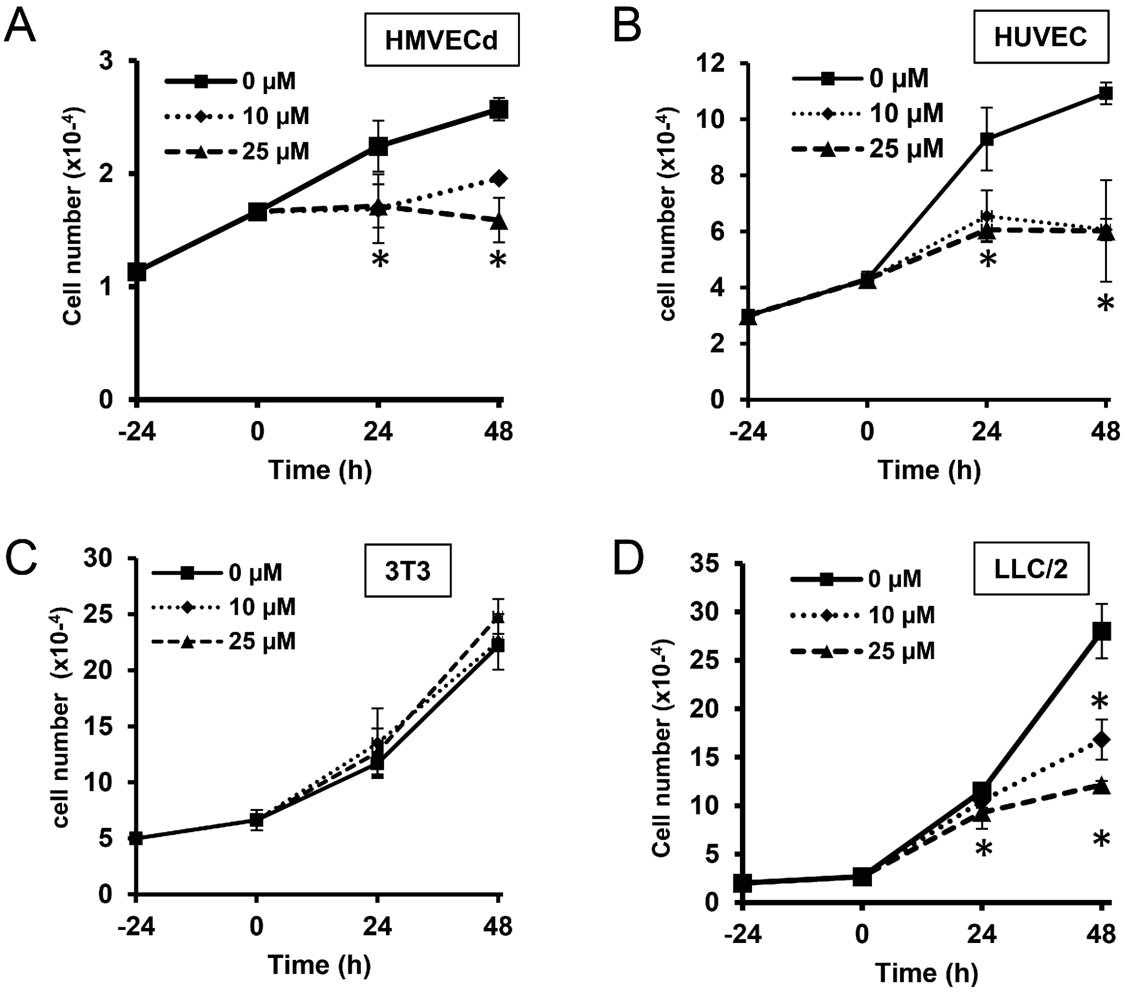

Figure S2: Growth curves of different cell types in the presence of COB223. Cells were seeded (respectively, 10^4 HMVECd cells in **(A)**, 20 x10^4 LLC/2 and HUVEC cells in **(B)**, and 50 x10^4 3T3 cells in **(D)**) and grown for 24 h under standard serum-supplemented culture conditions. 25 µM COB223 was then added and cell proliferation was measured by cell counting using the Millipore's Scepter™ automatic counter. The values represent means ± SD of triplicate determinations. * : $p < 0.05$

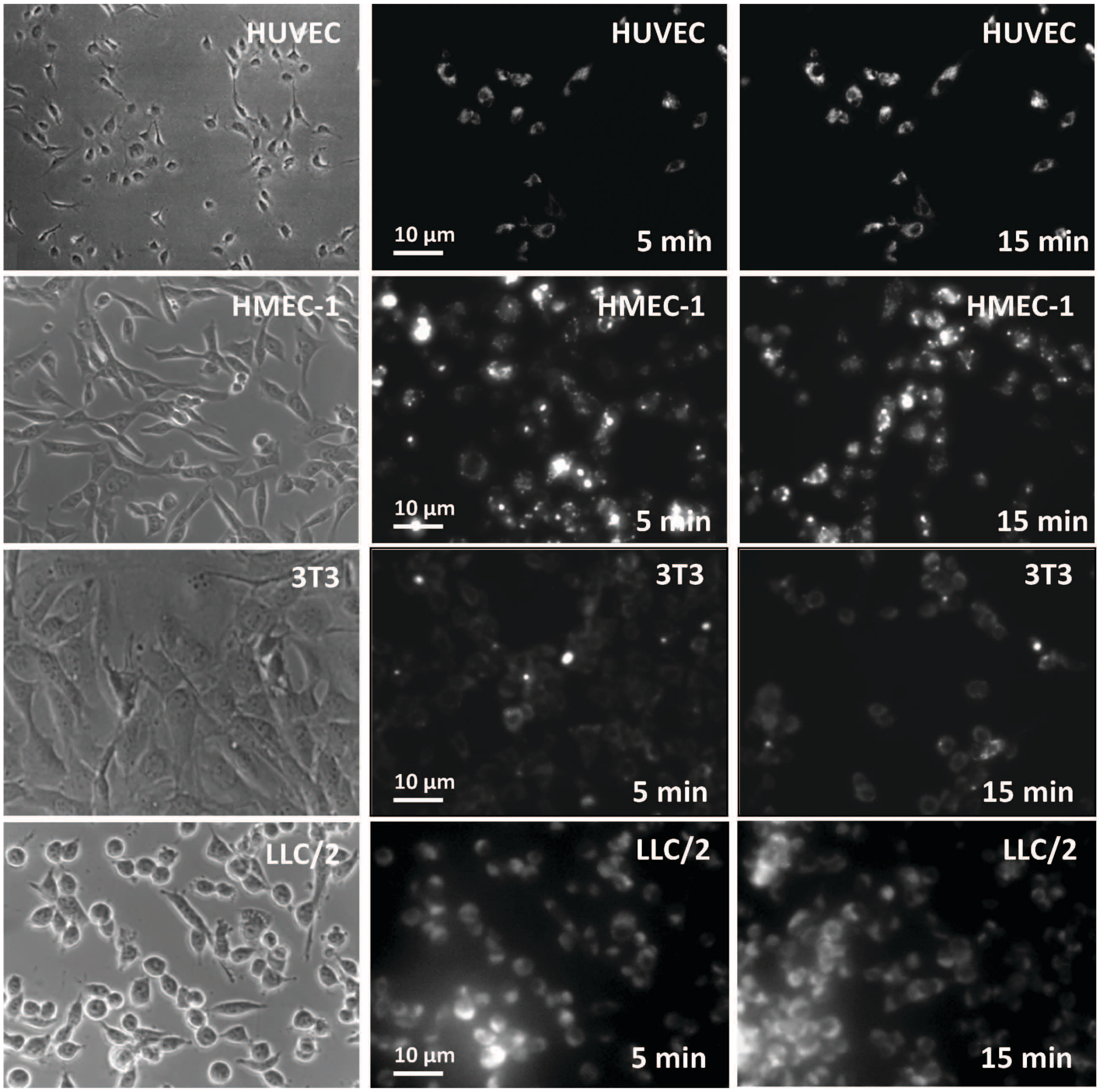

Figure S3: Cellular uptake of COB223. 25 µM of COB223 was added to HUVEC, HMEC-1, 3T3 or LLC/2 cells and its uptake was visualized after 5 (central column) and 15 minutes (right column) by fluorescent microscopy using a DAPI filter. All pictures were taken with the same exposure time. In the left column, pictures of distinct fields of the same cell lines were taken by phase contrast microscopy at the same magnification as the fluorescence pictures.
